# Polycystin-1 suppresses apoptotic signalling in endothelial cells and protects from atherosclerosis

**DOI:** 10.1093/cvr/cvag171

**Published:** 2026-08-10

**Authors:** Blanca Tardajos Ayllon, Julia Gras Font, Timothy A E Winks, Hannah Grimwood, Hannah Roddie, Mark Ariaans, Carl Wright, Timothy J A Chico, Maria Fragiadaki, Sheila E Francis, Albert C M Ong, Paul C Evans, Jovana Serbanovic-Canic

**Affiliations:** School of Medicine and Population Health, University of Sheffield, Sheffield S10 2RX, UK; William Harvey Research Institute, Barts and The London Faculty of Medicine and Dentistry, Queen Mary University of London, London, UK; School of Medicine and Population Health, University of Sheffield, Sheffield S10 2RX, UK; School of Medicine and Population Health, University of Sheffield, Sheffield S10 2RX, UK; School of Medicine and Population Health, University of Sheffield, Sheffield S10 2RX, UK; School of Medicine and Population Health, University of Sheffield, Sheffield S10 2RX, UK; School of Medicine and Population Health, University of Sheffield, Sheffield S10 2RX, UK; School of Medicine and Population Health, University of Sheffield, Sheffield S10 2RX, UK; School of Medicine and Population Health, University of Sheffield, Sheffield S10 2RX, UK; School of Medicine and Population Health, University of Sheffield, Sheffield S10 2RX, UK; William Harvey Research Institute, Barts and The London Faculty of Medicine and Dentistry, Queen Mary University of London, London, UK; School of Medicine and Population Health, University of Sheffield, Sheffield S10 2RX, UK; School of Medicine and Population Health, University of Sheffield, Sheffield S10 2RX, UK; William Harvey Research Institute, Barts and The London Faculty of Medicine and Dentistry, Queen Mary University of London, London, UK; School of Medicine and Population Health, University of Sheffield, Sheffield S10 2RX, UK

**Keywords:** Polycystin-1, Endothelial cells, Apoptosis, Atherosclerosis

## Abstract

**Aims:**

Polycystin-1 (*PKD1*) and -2 (*PKD2*) are causative genes for autosomal dominant polycystic kidney disease, which often presents with cardiovascular manifestations by mechanisms still not completely understood. PKD1 and PKD2 have been suggested to function as mechanoreceptors in endothelial cells (ECs), transducing mechanical forces exerted by the flowing blood into downstream signalling pathways.

**Methods and results:**

Our zebrafish functional screening of EC mechanoreceptors identified PKD1 and PKD2 as anti-apoptotic, protective factors in the zebrafish endothelium. In mice, we show that inducible EC-specific loss of PKD1, but not PKD2, led to increased atherosclerosis. To dissect the underlying mechanisms, we performed single cell RNA sequencing and identified candidate pathways regulating EC behaviour downstream from PKD1. Knockdown of *PKD1* in human aortic ECs resulted in increased EC apoptosis and decreased expression of athero-protective endothelial nitric oxide synthase, which was mediated by thrombospondin 1 and cellular communication network factor 1.

**Conclusion:**

By integrating *in vivo* and *in vitro* models with –omics approaches, we have identified PKD1 as a novel regulator of EC survival and protective factor against atherosclerosis development. We conclude that therapeutic targeting of this pathway may treat atherosclerosis.

**Time of primary review: 24 days**

## 1. Introduction

Atherosclerosis, the main cause of myocardial infarction and stroke, is a focal disease characterized by accumulation of lipids, inflammatory and apoptotic cells, extracellular matrix proteins and other material in branches, bends and bifurcations of arteries, forming atherosclerotic plaques. Athero-prone regions of arteries are characterized by disturbed flow (DF) with low magnitude of wall shear stress and spatial and temporal changes in flow direction, in contrast to athero-protected straight segments of arteries, where flow is undisturbed (UF) and laminar.^1,2^ Endothelial cells (ECs) sense and respond to wall shear stress via mechanoreceptors on their surface, which convert mechanical forces into downstream biochemical signalling pathways. A number of molecules and structures on ECs have been proposed to serve as mechanoreceptors, but this has been conclusively demonstrated for only a few of these, including piezo type mechanosensitive ion channel component 1 (PIEZO1), platelet and endothelial cell adhesion molecule 1 (PECAM1), plexin D1 (PLXND1) and integrin subunit beta 1 (ITGB1).^3–9^ In arterial regions exposed to athero-prone flow, ECs become activated with increased levels of inflammatory activation, apoptosis, proliferation, and endothelial to mesenchymal transition (EndMT), all of which contribute to plaque formation and atherosclerosis development and progression.^10^ Understanding how flow-induced mechanical forces link to downstream signalling pathways offers potential for targeting these molecules and has considerable therapeutic significance.

Mutations in either *PKD1* or *PKD2*, which encode for polycystin-1 (PKD1) and polycystin-2 (PKD2), respectively, cause autosomal dominant polycystic kidney disease (ADPKD). PKD1 and PKD2 have been proposed to function as mechanoreceptors in both endothelial and kidney epithelial cells.^11–13^ They have been postulated to form a hetero-oligomeric complex where PKD1 may function as a receptor for chemical and/or mechanical force stimulation, while PKD2 functions as a calcium ion channel.^13,14^ ADPKD, the most common hereditary kidney disease (1:400 to 1:1000 births),^15^ is characterized by development of fluid-filled kidney cysts, by mechanisms that are still not completely understood, but have been shown to involve deregulation of cell proliferation, survival, inflammatory activation, and autophagy.^16,17^ A number of different signalling pathways, many of which cross-talk, have been associated with ADPKD, including JAK/STAT, Wnt, mTOR, Hippo-YAP/TAZ, AMP-activated kinase, Ca^2+^ signalling, cyclic adenosine monophosphate and multiple growth factors including transforming growth factor beta (TGFβ).^18–22^ Notably, the disease is also accompanied by cardiovascular abnormalities, including hypertension, left ventricular hypertrophy, intra- and extra-cranial aneurysms and cardiac valvular defects.^23,24^ Indeed, cardiovascular manifestations represent the main cause of morbidity and mortality of ADPKD patients.^25^ Interestingly, endothelial dysfunction and increased carotid intima-media thickness have been observed in young, normotensive ADPKD patients, indicating an early cardiovascular involvement in ADPKD.^26,27^ It has recently been shown that endothelial PKD1 and PKD2 can regulate vascular tone in mice,^28–30^ however, the role of these two proteins in the development of atherosclerosis has not yet been elucidated.

In this study, for the first time, we have identified endothelial PKD1 as a regulator of EC survival with a protective effect against atherosclerosis development and revealed novel downstream targets of PKD1 in the endothelium, which mediate its effect on athero-protective signalling pathways.

## 2. Methods

### 2.1 Zebrafish

All zebrafish studies conformed to the institution’s ethical requirements, UK Home Office regulations and guidelines from Directive 2010/63/EU of the European Parliament on the protection of animals used for scientific purposes. Zebrafish care and experimental procedures were carried out under Project Licence 70/8588 issued by the UK Home Office. Maintenance, manipulation and staging of transgenic line Tg(*flk1:EGFP-NLS*)^31^ were carried out as described in standard husbandry protocols.^32,33^ ECs were isolated by fluorescence-activated cell sorting (FACS) from dissociated Tg(*flk1:EGFP-NLS*) embryos as described previously.^34^ Expression of candidate mechanoreceptors was measured using qPCR and gene-specific primers listed in Supplementary material online, *Table S1*. RNA probes for whole-mount *in situ* hybridization were generated using gene-specific primers listed in Supplementary material online, *Table S2* and whole-mount *in situ* hybridization was conducted following the protocol by Thisse *et al*.^35^ Morpholino antisense oligonucleotides (MOs) (GeneTools, LLC) were diluted in sterile water and ∼1 nL was injected into the yolk of a 1-4 cell stage embryo. Non-targeting control MO was used as a negative control. The list of MOs and their concentrations used in this study is presented in Supplementary material online, *Table S3*. The efficiency of MOs was validated by reverse transcriptase–PCR (RT–PCR) and qPCR. Total RNA was extracted from zebrafish embryos using RNeasy Mini Kit (QIAGEN) according to manufacturer’s protocol and 500 ng of total RNA was subjected to cDNA synthesis using iScript reverse transcriptase (Bio-Rad). Resulting cDNA was used as a template for PCR using gene-specific primers (listed in Supplementary material online, *Table S4*) and BioMix Red kit (Bioline) as per manufacturer’s instructions. The resulting RT–PCR products were analysed by agarose gel electrophoresis. Amplification of housekeeping gene beta-actin (*bact2*) was used as an internal control. Where no aberrant splicing or a decrease in wildtype band expression could be observed, sequencing of PCR products was carried out and knockdown validated using qPCR and wildtype-specific primers (see Supplementary material online, *Table S4*). Apoptotic cells in whole-mount zebrafish embryos were detected by active caspase-3 staining (see Supplementary material online, *Table S7*), as previously decribed.^36^ Imaging was performed using an Olympus FV1000 laser scanning confocal microscope with a 40× (numerical aperture (NA) 1.0) oil immersion objective. EC apoptosis was quantified by counting the total number of ECs (GFP^+^ cells; green) and the number of apoptotic ECs (GFP^+^ and active caspase 3^+^; yellow). The proportion of apoptotic cells was obtained by dividing the number of apoptotic cells by the total number of ECs.

### 2.2 Mice

All animal care and experimental procedures conformed to the institution’s ethical requirements, UK Home Office regulations and guidelines from Directive 2010/63/EU of the European Parliament on the protection of animals used for scientific purposes. Animal care and experimental procedures were carried out under Project Licence P5395C858 issued by the UK Home Office. Mice with conditional deletion of *Pkd1* or *Pkd2* in ECs (*Pkd1^iECKO^* and *Pkd2^iECKO^*) were generated through the crossbreeding of *Pkd1^fl/fl^* mice^37^ (kindly provided by Prof. David Huso, Johns Hopkins University, Baltimore, Maryland) and *Pkd2^fl/fl^* mice^38^ (obtained from JAX, stock #017292) with *Cdh5^Cre-ERT2^* mice.^39^ All mice were maintained on a C57BL/6J background. Genotyping was performed using PCR primers listed in Supplementary material online, *Table S5*. Cre activation was induced by intraperitoneal administration of tamoxifen (Sigma, T5648) in corn oil for five consecutive days (2 mg/mouse/day). Two weeks after the first injection of tamoxifen, hypercholesterolaemia was induced by intravenous tail vein injection of adeno-associated virus carrying a gain-of-function mutated version of proprotein convertase subtilisin/kexin type 9 (rAAV8-D377Y-mPCSK9) gene (Vector Core, North Carolina), followed by a high-fat diet (SDS UK, 829100) for 6 weeks, as previously detailed.^40^ Animals were sacrificed by a single intraperitoneal injection of sodium pentobarbital (800 mg/kg). Male mice aged between 1.5 and 3 months of age were used for experiments.

### 2.3 Mouse partial carotid ligation

The left carotid arteries of 6–8-weeks old C57BL/6J mice were partially ligated as described previously.^41^ Anaesthesia was induced and maintained using isoflurane by inhalation (induction: 3.5% isoflurane, 1 L/min oxygen; surgery: 1.5–3% isoflurane, 0.4 L/min oxygen) and the external, the internal and the occipital arteries were ligated allowing blood flow only via the superior thyroid artery. One week after the partial ligation, endothelial RNA was collected from the partially ligated left carotid arteries and control right carotid arteries from the same animals.

### 2.4 Endothelial RNA extraction from mouse arteries

Total RNA was extracted from mouse arterial ECs with QIAzol (79306, QIAGEN) and miRNeasy mini kit (217084, QIAGEN) using flushing method as described previously.^41,42^

### 2.5 Analysis of atherosclerotic plaques

Following euthanasia and perfusion-fixation with PBS and 4% paraformaldehyde (PFA), aortas were dissected, cleaned and stained with 0.5% Oil Red O solution in 60% isopropanol (Sigma) following the manufacturer’s protocol. Aortas were then imaged through a Stemi 2000 C microscope (Zeiss) using a Canon PowerShot A650 IS digital camera, and lesion areas were quantified using NIS elements analysis software (Nikon, NY).

### 2.6 Plasma lipid measurements

Blood samples collected by terminal cardiac puncture were subjected to centrifugation to obtain plasma. Plasma cholesterol levels (total, LDL, and HDL) and triglyceride levels were measured using a COBAS analyser.

### 2.7 *En face* staining of murine endothelium

Endothelial expression levels of specific proteins in mouse aortas were assessed by *en face* staining. Following euthanasia, aortae were perfused *in situ* with PBS and then perfusion-fixed with 4% PFA prior to harvesting. Fixed aortae were tested by immunostaining using antibodies listed in Supplementary material online, *Table S7*. ECs were identified by co-staining using an anti-CDH5 antibody (see Supplementary material online, *Table S7*). Nuclei were identified using TO-PRO-3 (ThermoFisher). Stained vessels were mounted in ProLong Gold (ThermoFisher) prior to visualization of endothelial surfaces *en face* using confocal microscopy (Olympus FV1000 confocal microscope). Isotype controls and omission of the primary antibody were used to control for non-specific staining. Protein expression levels were assessed by quantification of the mean fluorescence intensities using Fiji software.

### 2.8 Immunohistochemistry

Mice were euthanized and both kidneys and aortic roots were removed and submerged in 4% PFA overnight, before being transferred to PBS. The kidneys were bisected and processed into paraffin blocks, showing the internal face of the tissues, with sections then cut at 5 µm intervals. Slides were dewaxed in xylene before being transferred to Weigerts Haematoxylin for 10 min then washed in tap water until clear. Sections were then stained in Picrosirius Red for an hour, washed in acidified water until all excess red is removed, and dehydrated prior to mounting with DPX. Slides were imaged on an Echo Rebel microscope at 4× magnification and analysed for relative surface of collagen against total tissue surface area using Fiji’s colour threshold function. For alpha-smooth muscle actin staining (ACTA2), slides were dewaxed in xylene and blocked in 10% goat serum for 30 min, before incubation with rabbit anti-ACTA2 at 1:500 dilution, overnight at 4°C. Primary antibody was washed off with PBS and probed with HRP-conjugated Goat anti-rabbit secondary antibody (Dako, P0448) for 2 h at room temperature. The excess secondary antibody was washed off with PBS then the slides were developed with DAB Substrate Kit (Vector Labs, SK-4100) for 5 min. Slides were counterstained for 25 s in haematoxylin and Scott’s Tap Water Substitute, and then mounted with DPX. Images of arteries of a similar size, from the 10× magnification were analysed for media thickness using Fiji. Click-iT™ TUNEL Colorimetric IHC Detection Kit (Invitrogen) was used to detect apoptosis in cross sections of aortic roots, following manufacturer’s instructions.

### 2.9 Tissue processing and FACS for scRNA-seq

The left ventricle of the murine heart was perfused with heparin sodium (20 U/mL) in PBS. Aortas from age- and sex-matched male *Pkd1^iECKO^* and control mice (*Pkd1^fl/f^ Cdh5^+/+^*) were dissected from the aortic root to below the iliac artery. After removing fat and connective tissue, aortas were transferred into ice-cold PBS. To generate single-cell suspensions, the isolated aorta was incubated in collagenase type I (450 U/mL), collagenase type XI (125 U/mL), hyaluronidase type 1-s (60 U/mL), deoxyribonuclease I (60 U/mL), and elastase (0.5 mg/mL) in PBS for 10 min at 37°C to allow for the separation of the adventitia cell layer. After removing the adventitia, tissue was divided into 2-mm pieces and further incubated in the enzymatic solution for 1 h at 37°C to produce a single-cell suspension. The cell suspension was then filtered through a 40-µm cell strainer, washed, and resuspended in 1% bovine serum albumin (BSA)–PBS. For the remainder of the procedure, cells were kept on ice. Single-cell suspensions were incubated for 5 to 10 min with TruStain FcX™ PLUS (anti-mouse CD16/32) antibody (BioLegend) to block non-specific binding of immunoglobulin to Fc receptors, before staining with allophycocyanin-conjugated anti-CD45 and Alexa Fluor 488–conjugated anti-CD31 antibodies. To exclude dead cells, samples were stained with TO-PRO-3. Stained aortic cells were washed with 1% BSA-PBS buffer before analysis, and CD31^+^, CD45^−^, and TO-PRO-3^−^ cells were sorted into 384-well plates containing reverse transcription primers, deoxyribonucleotide triphosphates, External RNA Controls Consortium (ERCC) RNA Spike-Ins, and mineral oil using a BD FACSMelody cell sorter (BD Biosciences). Plates containing capture cells were immediately stored at −80°C before sequencing.

### 2.10 Single-cell RNA sequencing and bioinformatics

scRNA-seq libraries were generated using the SORT-seq protocol,^43^ a partially robotized method based on the CEL-Seq2 protocol^44^ as described previously.^45^ Briefly, single cells were lysed at 65°C for 5 min, and second-strand mixers and reverse transcriptase were then added to the wells using the Nanodrop II liquid handling platform (GC Biotech). After reverse-transcribing the mRNA of each cell, double-stranded complementary DNAs (cDNAs) from single cells were pooled, and *in vitro* transcription was performed for linear amplification, which resulted in amplified RNA. TruSeq small RNA primers (Illumina) were used to prepare the Illumina sequencing libraries, and these DNA libraries were then sequenced paired-end at 75–base pair read length using the Illumina NextSeq (performed commercially by Single Cell Discoveries, Utrecht, the Netherlands). Both the RNA yield of the amplified RNA and the quality and concentration of the final cDNA libraries were assessed by Bioanalyzer (Agilent). Cells were filtered using standard per-cell QC metrics, excluding cells with low complexity or evidence of poor quality (genes per cell <300, transcripts per cell <1,000, mitochondrial gene fraction >16%). Dimensional reduction and clustering of our scRNA-seq dataset were performed using the R package Seurat (v3.1). Differential expression analysis was performed between clusters, and marker genes for each cluster were determined with the Wilcoxon rank-sum test with *P* value<0.001 and a minimum log-fold change threshold of 0.25 using Seurat and BBrowser (version 2.10.10). Differential expression analysis, tSNE representations showing the expression of defined gene sets and expression of single transcripts on the tSNE embedding were performed using the software BBrowser (version 2.10.10).

### 2.11 EC culture and exposure to flow

Human aortic endothelial cells (HAoECs) were purchased from PromoCell (lot numbers presented in Supplementary material online, *Table S9*) and cultured as per the manufacturer’s instructions. Experiments were performed using cells from multiple individual donors that were not pooled and cells at passages 3–5 were used for the experiments. For flow experiments, HAoECs were seeded onto ibidi μ-Slides I^0.4^ (Luer ibiTreat, ibidi) and used when fully confluent. Flowing medium was then applied using the ibidi pump system to generate high laminar (13 dyne/cm^2^), low laminar (4 dyne/cm^2^) or low oscillatory wall shear stress. For low oscillatory wall shear stress, HAoECs were exposed to a repeated cycle of 2 h of oscillatory flow (±4 dyne/cm^2^, 0.5 Hz), followed by 10 min of unidirectional flow (4 dyne/cm^2^), to ensure redistribution of nutrients. The slides and pump apparatus were placed in a cell culture incubator at 37°C.

### 2.12 Gene silencing

HAoECs were transfected with siRNA sequences targeting *PKD1* (ON-TARGETplus Human *PKD1* siRNA, L-007666-00, Horizon Discovery), *THBS1* (ON-TARGETplus Human *THBS1* siRNA, L-019743-00, Horizon Discovery), *CCN1* (ON-TARGETplus Human *CYR61* siRNA, L-004263-00, Horizon Discovery) and *PKD2* (ON-TARGETplus Human PKD2 siRNA, L-006288-02, Horizon Discovery) using Lipofectamine™ RNAiMAX Transfection Reagent (ThermoFisher), following the manufacturer’s instructions. Non-targeting siRNA sequences served as a negative control (ON-TARGETplus Non-targeting Control Pool, D-001810-01, Horizon Discovery). Final concentration of 10 nM siRNA was used for the experiments.

### 2.13 Immunofluorescence staining of cultured HAoEC

HAoECs were fixed with 4% PFA and permeabilized with 0.1% Triton X-100 in PBS. Following blocking with 5% goat serum in PBS for 1 h, monolayers were incubated overnight at 4°C with primary antibodies against active caspase-3 (apoptosis marker) and CDH5 (endothelial marker) (see Supplementary material online, *Table S7*). This was followed by incubation with appropriate AlexaFluor488- or Alexafluor568-conjugated secondary antibodies (ThermoFisher) for 2 h at room temperature. Nuclei were identified using 2 µg/mL DAPI (Sigma). Images were taken with a wide-field fluorescence microscope (Leica DMI4000B) and analysed using Fiji software to calculate the frequency of active caspase-3 positive cells. Isotype controls and omission of the primary antibody were used to control for non-specific staining.

### 2.14 Quantitative PCR

Total RNA was extracted using RNeasy Mini Kit (74104, QIAGEN) and reverse transcribed into cDNA using the iScript cDNA synthesis kit (1708891, Bio-Rad), following the manufacturers’ protocols. Quantitative real-time PCR (qPCR) was used to assess the levels of transcripts with gene-specific primers (see Supplementary material online, *Tables S1* and *S6*). Reactions were prepared using SsoAdvanced universal SYBR®Green supermix (172-5271, Bio-Rad) and following the manufacturer’s instructions, and were performed in triplicate. Expression values were normalized against the house-keeping gene (zebrafish *bact2*, mouse *Hprt*, and human *HPRT*). Fold differences were calculated using the ΔΔCt method.

### 2.15 Assay of permeability

The permeability of HAoEC monolayers was determined using rhodamine (Rd)-labelled albumin and Transwell inserts, as previously described.^46^ HAoECs were cultured in 6-well Transwell inserts (CC401, Corning) and incubated for 72 h following transfection with gene-specific siRNAs. The media in the upper compartment was then replaced with media containing 1% BSA and Rd-labelled albumin (1 mg/mL). Media in the lower compartment was sampled after 1 h and fluorescence was measured using a fluorimeter (Varioskan, Thermoscientific) with excitation at 570 nm and emission at 600 nm. Rd-albumin concentration was calculated using a standard curve.

### 2.16 Nitric oxide assay

Concentration of nitric oxide (NO) in the cell culture media was measured using colorimetric Nitric Oxide Assay Kit (Sigma Aldrich), following manufacturer’s protocol. It is designed to accurately measure NO production following reduction of nitrate (NO_3_^−^) to nitrite (NO_2_^−^) using an improved Griess method. Media was collected 72 h following the transfection of HAoEC with *PKD1* siRNA or negative control siRNA.

### 2.17 Western blotting

Total cell lysates were isolated using lysis buffer (containing 2% SDS, 10% Glycerol and 5% β-mercaptoethanol). Western blotting was carried out using specific antibodies against PDHX (housekeeper) and endothelial nitric oxide synthase (eNOS) (see Supplementary material online, *Table S7*). Horseradish peroxidase-conjugated secondary antibodies were obtained from Dako. Chemiluminescent detection was carried out using ECL Select™ Western Blotting Detection Reagent (Cytiva). Membranes were imaged using the Gel Doc XR+ system (Bio-Rad). Relative protein quantification was performed using Image Lab Software (Bio-Rad).

### 2.18 Lactate dehydrogenase assay

Cell death was measured using CyQUANT lactate dehydrogenase (LDH) Cytotoxicity Assay (ThermoFisher) and following manufacturer’s protocol. Media samples were collected on the day of transfection and 1, 2, and 3 days following transfection of HAoECs with gene-specific siRNAs. The absorbance was measured by Hidex Sense microplate reader (Hidex) at 490 and 680 nm.

### 2.19 Statistical analysis

Data are presented as mean values ± standard error of the mean. Statistical analyses were performed using GraphPad Prism software. A Shapiro–Wilk normality test was performed on non-normalized data prior to performing the non-parametric tests. The specific test used is detailed in the figure legend.

## 3. Results

### 3.1 Functional screening of endothelial mechanoreceptors in zebrafish identifies PKD1 and PKD2 as regulators of EC apoptosis

To identify new flow-sensitive regulators of EC apoptosis in response to blood flow, we used our previously established zebrafish model.^47^ We selected 12 known and putative EC mechanoreceptors and identified their orthologues in zebrafish (see Supplementary material online, Figure  *S1A*). The candidate genes were chosen to represent all major mechanoreceptor classes demonstrated to mediate endothelial responses to shear stress, including ion channels (PIEZO1, PKD2, and TRPV4), adhesion molecules (CDH5 and PECAM1), integrins (ITGB1 and ITGB5), G-protein coupled receptors (BDKRB2), proteoglycans (GPC1 and SDC4), ciliary components (PKD1 and PKD2), glycocalyx components (SDC4), and caveolar proteins (CAV1) (see Supplementary material online, Figure  *S1A*).^2,7,48^ The majority of selected human genes had one corresponding zebrafish orthologue (*BDKRB2, CAV1, CDH5, ITGA5, PECAM1, PIEZO1, PKD2, SDC4*, and *TRPV4*), while some had 2 (*GPC1* and *PKD1*) or 4 zebrafish orthologues (*ITGB1*). To determine whether the selected genes are expressed in the endothelium of zebrafish embryos, we isolated ECs from 30 h post-fertilization (hpf) Tg(*flk1:EGFP-NLS*) embryos by FACS and performed gene expression analysis using qPCR (see Supplementary material online, Figure  *S1B–C*). We found *cav1, cdh5, itga5, itgb1a, itgb1b, itgb1b.1, pecam1, piezo1, pkd1a, pkd2*, and *sdc4* to be expressed in zebrafish ECs, while *bdkrb2, gpc1a, gpc1b, itgb1b.2, pkd1b*, and *trpv4* showed no EC expression at 30 hpf (see Supplementary material online, Figure  *S1C*). For human genes with more than one zebrafish orthologue which showed expression in zebrafish ECs (*ITGB1* and *PKD1*), differential gene expression between different paralogues was confirmed by whole-mount *in situ* hybridization (see Supplementary material online, Figure  *S1D*). Based on their expression in ECs, we selected *cav1, cdh5, itga5, pecam1, piezo1, pkd1a, pkd2*, and *sdc4* for the functional screening study (*Figure 1A*). *ITGB1* was excluded from subsequent studies due to potential functional redundancy between the 3 paralogues that were found to be expressed in zebrafish ECs (see Supplementary material online, Figure  *S1D*).

**Figure 1:**
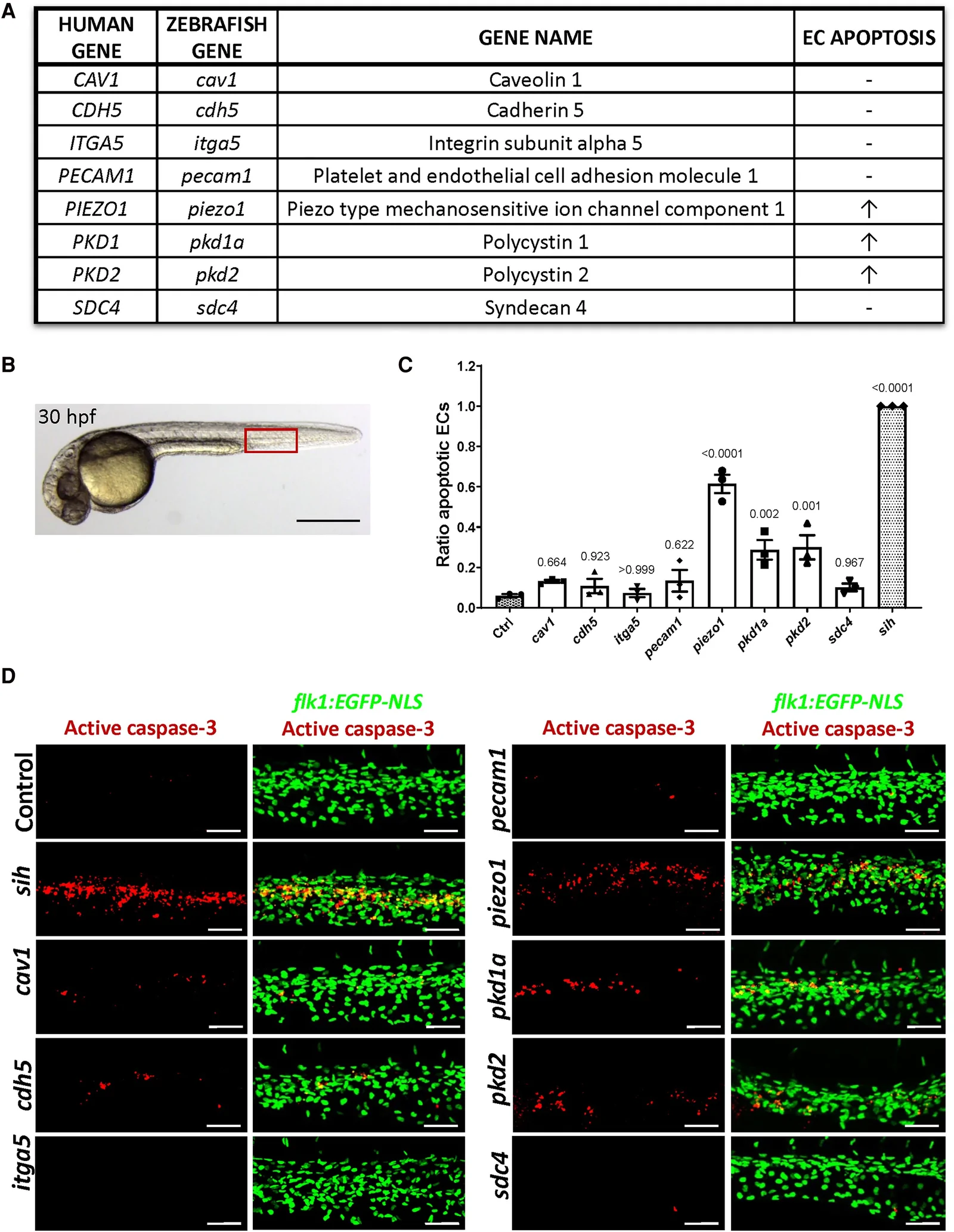
Functional screening of endothelial mechanoreceptors in zebrafish embryos to identify regulators of endothelial apoptosis. (*A*) Table showing putative and confirmed EC mechanoreceptors selected for the functional screening based on their confirmed expression in zebrafish endothelium at 30 h post-fertilization (hpf). (*B*) Zebrafish embryo at 30 hpf. The boxed region represents the region that is studied in (*C* and *D*). Zebrafish transgenic Tg(*flk1:EGFP-NLS*) embryos, in which EC nuclei are labelled, were injected with MO targeting *cav1, cdh5, itga5, pecam1, piezo1, pkd1a, pkd2*, or *sdc4*. Embryos lacking blood flow injected with *silent heart* (*sih*) MO were used as a positive control. Non-targeting control MO was used as a negative control. EC apoptosis was studied by whole-mount active caspase-3 staining of 30 hpf embryos, with apoptotic ECs identified by co-localisation of active caspase-3 signal with labelled EC nuclei. (*C*) The proportion of apoptotic ECs (number of apoptotic ECs divided by the total number of ECs) normalized to *sih* MO-injected embryos was calculated and is presented in the graph. Each data point represents an independent experiment with *n* ≥ 5 embryos. Differences between groups were analysed using a one-way ANOVA with Dunnett’s *post-hoc* test vs. control embryos and *P* values are shown in the graph. (*D*) Whole-mount active caspase-3 staining of 30 hpf embryos. Lateral view, anterior to the left, dorsal up. Scale bars: (*B*) 50 µm; (*D*) 500 µm.

We next knocked down candidate gene expression in Tg(*flk1:EGFP-NLS*) embryos using morpholino oligonucleotide (MO)-mediated gene silencing and assessed the effect of gene knockdown on EC apoptosis using active caspase-3 staining at 30 hpf (*Figure 1B–D*). The ability of MOs to down-regulate target gene expression was validated by PCR (see Supplementary material online, Figure  *S2*) and qPCR (see Supplementary material online, Figure  *S3*). Aberrant splicing and/or decrease in expression of wildtype transcript was detected for splice-blocking MOs targeting *itga5*, *pecam1*, *pkd1a*, and *sdc4* (see Supplementary material online, Figure  *S2*). Since *cav1* and *cdh5* MOs did not show a significant reduction using standard PCR and agarose gel electrophoresis (see Supplementary material online, Figure  *S2*), successful knockdown was validated following sequencing of PCR products and assessment of gene expression using qPCR and primers spanning the MO-targeted exon–exon junction (see Supplementary material online, Figure  *S3*). For *piezo1* and *pkd2*, previously validated translation-blocking MOs were used^49,50^ and, as expected, *pkd2* MO-injected embryos showed the characteristic ‘curly up’ phenotype (*Figure 1D*).^51^ As a positive control, we used *silent heart* (*sih*) embryos lacking blood flow due to knockdown of cardiac troponin T2, which we previously established to exhibit increased levels of EC apoptosis at 30 hpf (*Figure 1C* and *D*).^47^ Knockdown of *piezo1, pkd1a* and *pkd2* significantly increased EC apoptosis in zebrafish embryos (10.36-fold, *P* < 0.0001; 4.84-fold, *P* = 0.002; 5.05-fold, *P* = 0.001 vs. control embryos, respectively), while *cav1, cdh5, itga5, pecam1*, or *sdc4* were not required for flow-mediated EC survival (*Figure 1C* and *D*). Knockdown of *piezo1*, *pkd1a*, or *pkd2* in the absence of blood flow (*sih* embryos) did not have an additive effect on EC apoptosis, suggesting that the effect of these three genes on EC survival is flow-dependent (see Supplementary material online, Figure  *S4*).

These results indicated an important role for PIEZO1 and polycystins (PKD1 and PKD2) in regulating EC survival, which has important implications for the development and progression of cardiovascular diseases, such as atherosclerosis. PIEZO1 has already been reported to play a complex role in atherosclerosis development, depending on the wall shear stress conditions: it is athero-protective under UF flow, while it promotes atherosclerosis under DF conditions.^52,53^ On the other hand, the role of PKD1 and PKD2 in atherosclerosis has not yet been studied. We therefore next set out to test the role of endothelial PKD1 and PKD2 in atherosclerosis development using the murine model.

### 3.2 Endothelial polycystin-1, but not polycystin-2, protects from experimental atherosclerosis development in mice

To test whether expression of *Pkd1* and *Pkd2* is regulated by wall shear stress, we measured their expression in partially ligated mouse carotid arteries (*Figure 2A* and *B* and Supplementary material online, Figure  *S7A*). *Pkd1* and *Pkd2* mRNA expression were significantly induced in the partially ligated left carotid arteries with athero-prone flow (2.43-fold, *P* = 0.0099 and 2.92-fold, *P* = 0.039, respectively), compared with control right carotid arteries with UF from the same animals. This indicated that *Pkd1* and *Pkd2* mRNA expression is up-regulated by atherogenic flow in mouse arteries.

**Figure 2:**
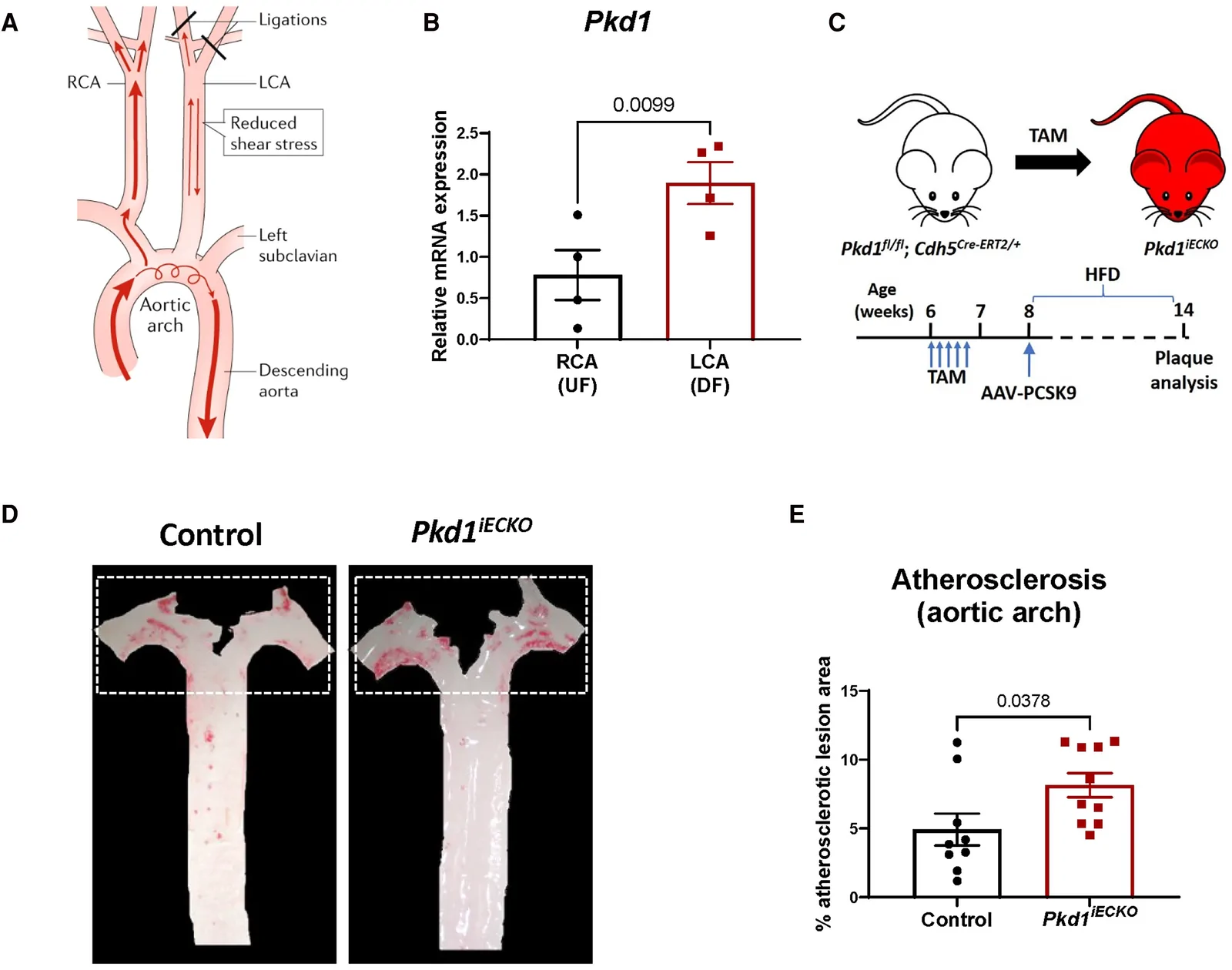
Endothelial PKD1 is flow-responsive and protects from atherosclerosis development in the mouse model. (*A* and *B*) *Pkd1* mRNA expression is induced by DF conditions. (*A*) Schematic representation of the partial ligation model in which three of the four branches of the left carotid artery (LCA) are ligated, leading to a reduction in shear stress and DF in the LCA compared with the right carotid artery (RCA) with UF. Taken from Souilhol *et al*.^10^ and adapted from Muto *et al*.^54^ CC BY 4.0. (*B*) C57BL/6 mice at 8 weeks of age (*n* = 4) were subjected to partial ligation of the LCA, whereas the RCA was sham-operated as a baseline control. Intimal RNA from LCA and RCA was collected 2 days after surgery. Relative *Pkd1* mRNA expression was measured by qPCR. (*C*-*E*) Endothelial PKD1 protects from atherosclerosis development. (*C*) Timeline of *Pkd1* deletion in a mouse model of hypercholesterolaemia. *Pkd1^fl/f^ Cdh5^CreERT2/+^* (*Pkd1^iECKO^*) mice aged 6 weeks and littermates lacking Cre (*Pkd1^fl/fl^ Cdh5^+/+^*; controls) received five intraperitoneal injections of tamoxifen (TAM) and one intravenous injection of AAV-PCSK9 virus at specified time points. After 6 weeks of feeding with high-fat diet (HFD), the mice were culled, and plaque area was quantified. (*D*) Representative images of aortas isolated from *Pkd1^iECKO^* and control mice stained with Oil Red O. Aortic arch is indicated by white dashed line. (*E*) Quantification of plaque burden in the aortic arch was determined by calculating the percentage of aortic surface area covered by plaque for *Pkd1^iECKO^* mice (*n* = 10) and littermate controls (*n* = 9). (*B* and *E*) Differences between groups were analysed using paired *t*-test (*B*) and unpaired *t*-test (*E*), with *P* values shown in the graphs. Scale bar: B, 20 µm.

To dissect the roles of endothelial *Pkd1* and *Pkd2* in atherosclerosis, we deleted *Pkd1* or *Pkd2* from the mouse endothelium using *Cdh5^Cre-ERT2/+^* line crossed with *Pkd1^fl/fl^* or *Pkd2^fl/fl^* lines, respectively. This resulted in generation of *Pkd1^iEC-KO^* or *Pkd2^iEC-KO^* mice and control mice (*Figure 2C*). Successful deletion of *Pkd1* or *Pkd2* in ECs following tamoxifen treatment was validated by gene expression analysis of endothelial RNA extractions, showing >80% efficiency of Cre-dependent gene deletion in ECs (see Supplementary material online, Figure  *S5*). At the same time, there was no difference in *Pkd1* or *Pkd2* mRNA expression in RNA extracted from the vascular smooth muscle cell (VSMC)-enriched aortic samples from *Pkd1^iEC-KO^* or *Pkd2^iEC-KO^* compared with control mice, respectively, confirming that deletion is specific to ECs (see Supplementary material online, Figure  *S5D*, *H*).

To generate atherosclerotic lesions, the mice were treated with AAV-PCSK9 to induce hypercholesterolaemia, followed by feeding with a high-fat diet for 6 weeks (*Figure 2C*). Endothelial deletion of *Pkd1* or *Pkd2* had no significant effect on body weight or plasma cholesterol and triglyceride levels compared with respective control animals (see Supplementary material online, Figure  *S6B–F* and Figure *S7E–I*). Analysis of atherosclerotic plaques of *Pkd1^iEC-KO^* mice showed significantly increased lesion area in the aortic arch (1.66-fold vs. control, *P* = 0.0378), which is an athero-prone site, but not in the athero-protected descending aorta, compared with littermate control animals (*Figure 2D* and *E* and Supplementary material online, Figure  *S6A*). In contrast, *Pkd2^iEC-KO^* mice did not show a significant difference in atherosclerotic plaque formation compared with controls (see Supplementary material online, Figure  *S7B–D*). In agreement with previous findings, *Pkd1^iEC-KO^* mice did not show development of cysts in their kidneys.^30^ Concurrently, *Pkd1^iEC-KO^* mice did not show signs of hypertension, as indicated by investigation of renal fibrosis and thickness of medium sized arteries (see Supplementary material online, Figure  *S8A–D*). While we cannot exclude subtle blood pressure changes, no structural or fibrotic changes indicative of sustained hypertension were observed. Therefore, our data indicate that endothelial PKD1 protects against atherosclerosis development at the athero-prone sites.

To investigate the effect of PKD1 on endothelial survival in mice, we assessed EC apoptosis in the aortic roots of atherosclerotic *Pkd1^iEC-KO^* mice and controls (see Supplementary material online, Figure  *S8E–F*). Endothelial deletion of *Pkd1* resulted in increased apoptosis of ECs overlying the plaques (7% apoptosis in knockouts vs. 2.13% in controls; *P* = 0.038). These results suggest that, similarly to our findings in zebrafish, PKD1 is required for EC survival *in vivo* and, additionally, that PKD1 protects from EC apoptosis during atherosclerosis development.

### 3.3 Polycystin-1 promotes athero-protective, anti-apoptotic endothelial subsets in mouse aortic ECs

To elucidate the mechanisms underlying athero-protective role of PKD1 in the endothelium, we performed single cell RNA sequencing (scRNAseq) of ECs isolated from aortas of *Pkd1^iEC-KO^* and control mice. The scRNA-seq profiles of 887 cells from four experiments which passed stringent quality control were analysed. CD31^+^CD45^−^TO-PRO-3^−^ cells were isolated by FACS and processed for scRNAseq (see Supplementary material online, Figure  *S9*). Sort-seq analysis of isolated cells identified six distinct clusters (*Figure 3A*), of which clusters 1-4 and 6 consistently expressed multiple EC markers, whereas cluster 5 showed enriched expression for VSMC markers and was therefore removed from analysis of EC heterogeneity (see Supplementary material online, Figure  *S10*, heatmap; Supplementary material online, Figure  *S11*, marker expression plots). Notably, expression levels of VSMC markers in endothelial clusters were markedly lower than in cluster 5, further supporting their endothelial identity. Consistent with this, clusters 1-4 and 6 are closely similar to previously published EC clusters from the murine aorta.^45,55,56^

**Figure 3:**
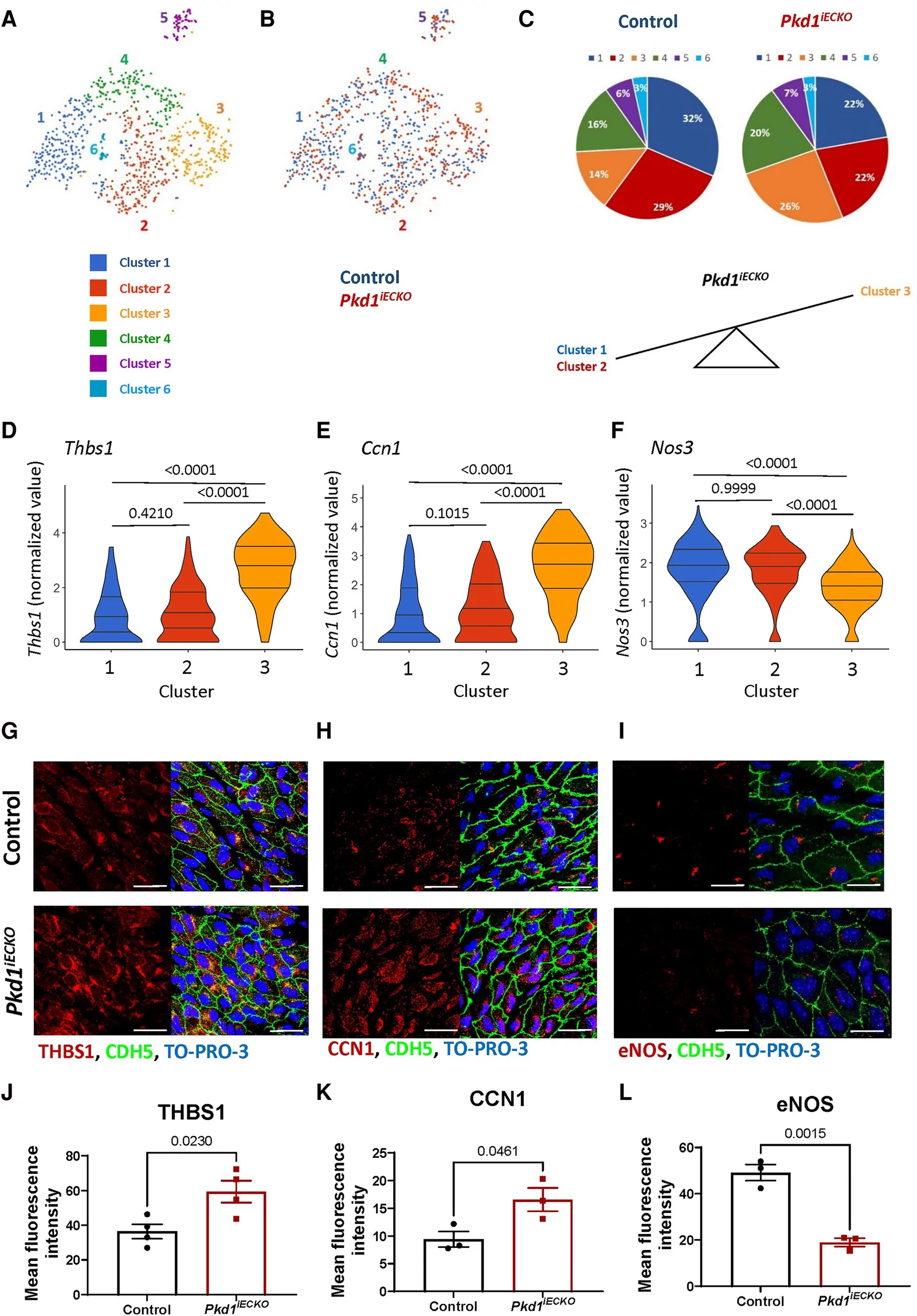
Single cell RNA sequencing analysis of *Pkd1*-deficient mouse aortic ECs and validation of PKD1 downstream targets. (*A-F*) *Pkd1^fl/fl^ Cdh5^Cre-ERT2/+^* or *Pkd1^fl/fl^ Cdh5^+/+^* littermate controls were injected with tamoxifen for five consecutive days to activate Cre and generate *Pkd1^iECKO^* and control mice, respectively. Two weeks later, aortas from *Pkd1^iECKO^* and control mice were processed to generate single cells and then analysed by FACS of CD31^+^ CD45^−^ cells coupled to scRNAseq. (*A*) tSNE representation of single-cell transcriptomes from *Pkd1^iECKO^* and control mice, with cells grouped according to cluster assignment. Clusters were identified using unbiased hierarchical clustering. (*B*) tSNE showing the cell contribution of *Pkd1^iECKO^* and control cells to each subpopulation. (*C*) Pie chart representation of cell distribution across cell clusters in *Pkd1^iECKO^* and control mice (top). Graphic representation of relative contribution of clusters 1, 2, and 3 to *Pkd1^iECKO^* ECs, compared with control. (*D-F*) Violin plots indicating expression distribution of *Thbs1* (*D*), *Ccn1* (*E*), and *Nos3* (*F*) in clusters 1, 2, and 3 identified by single cell RNA sequencing of *Pkd1*-deficient and control mouse aortic ECs. (*G-I*) Aortic arches were isolated from 8 week-old *Pkd1^iECKO^* and control mice and *en face* immunostainings were performed using antibodies against THBS1 (*G*), CCN1 (*H*), or eNOS (*I*). Endothelium was co-stained with anti-CDH5 antibody and nuclei were detected using TO-PRO-3. Images show inner curvature, athero-prone regions of the aortic arches. (*J-L*) Quantification of relative protein expression in the inner curvature of the aortic arches expressed as mean fluorescence intensity. *N* = 3–4 mice per group. Differences between groups were analysed using unpaired *t*-test with *P* values shown in the graphs. Scale bars: (*G*-*I*) 20 µm.

Endothelial clusters 4 and 6 showed similar enrichment in control and *Pkd1^iEC-KO^* ECs (*Figure 3B* and *C*). Interestingly, cluster 3 was expanded in *Pkd1^iEC-KO^* ECs (26% of total *Pkd1^iEC-KO^* ECs) compared with control ECs (14% of total control ECs), at the expense of clusters 1 and 2. In cluster 3, 66% of cells were *Pkd1^iEC-KO^* ECs and 34% were control ECs (*Figure 3B* and *C*). Functional annotation revealed diverse functions associated with *Pkd1*-regulated cluster 3, including the following terms: Regulation of apoptotic signalling pathway, Positive regulation of cell death and Fluid shear stress and atherosclerosis (see Supplementary material online, Figure  *S12A*).

We selected for validation four potential PKD1 downstream targets in the endothelium: thrombospondin 1 (THBS1), cellular communication network factor 1 (CCN1), bone morphogenetic protein 4 (BMP4) and thioredoxin domain containing 5 (TXNDC5). These were among top 20 most up-regulated genes in *Pkd1*-regulated cluster 3, compared with clusters 12, 4, and 6 and they were selected based on the following: (i) Known role in regulating EC apoptosis; (ii) Known role in promoting atherosclerosis; (iii) Inhibitory effect on NO signalling (*Figure 3D* and *E* and Supplementary material online, Figure  *S12B–C*).^57–68^ We also examined eNOS, due to implied involvement of NO signalling in *Pkd1*-downstream effects (*Figure 3F* and Supplementary material online, Figure  *S12B*). To test whether the selected molecules are differentially regulated in *Pkd1*-deficient ECs at the protein level, we examined their expression using *en face* immunostaining in *Pkd1^iEC-KO^* and control mice. Aortas from *Pkd1^iECKO^* mice showed significant increase in protein expression of THBS1 (1.63-fold, *P* = 0.023) and CCN1 (1.76-fold, *P* = 0.046) compared with controls, but not BMP4 and TXNDC5, in the athero-prone inner curvature (*Figure 3G*, *H, J,* and *K* and Supplementary material online, Figure  *S13A–F*). Additionally, we found reduced expression of eNOS in the aortas of *Pkd1^iEC-KO^* mice compared with control mice (0.38-fold, *P* = 0.0015) (*Figure 3I, L*). These results indicate that THBS1 and CCN1 may be downstream targets of PKD1 in ECs and that they may modulate PKD1-dependent eNOS signalling.

### 3.4 PKD1 exerts its protective effect in human ECs via THBS1 and CCN1

To test whether PKD1 plays a conserved anti-apoptotic role in human ECs, we knocked down its expression in HAoECs using siRNAs. Successful knockdown was confirmed by qPCR and it showed 86% decrease in *PKD1* mRNA expression in *PKD1* siRNA-transfected cells compared with control-transfected cells (*Figure 4A*). *PKD1*-deficient ECs showed significantly increased levels of apoptosis (1.12 ± 0.10% *PKD1* siRNA vs. 0.62 ± 0.11% control siRNA, *P* = 0.0139), as demonstrated by active caspase-3 staining (*Figure 4B* and *C*). In contrast, *PKD2* knockdown, which was approximately 85% efficient, did not result in a significant change in EC apoptosis (see Supplementary material online, Figure  *S14*).

**Figure 4:**
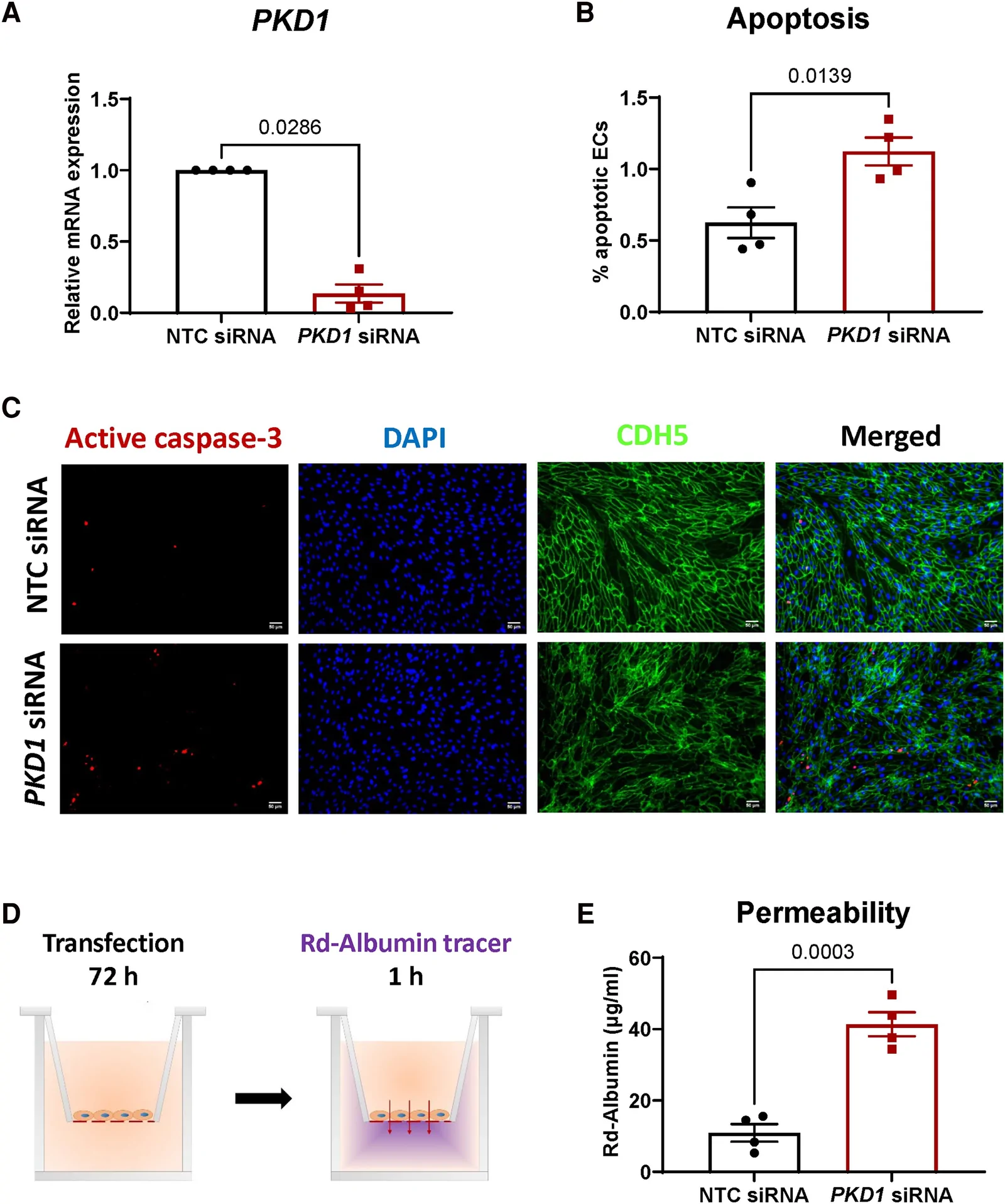
Knockdown of *Pkd1* in HAoECs results in increased levels of apoptosis and permeability to Rd-labelled albumin. (*A-C*) HAoECs were treated with 10 nM *PKD1* siRNA or with non-targeting control (NTC) siRNA for 72 h. (*A*) The efficiency of *PKD1* siRNA was assessed by qPCR (*n* = 4 donors) using *HPRT* as a housekeeping gene. EC apoptosis was assessed by immunostaining using anti-active caspase-3 antibody and co-staining with EC marker CDH5 and DAPI. (*B*) Quantification of EC apoptosis in *PKD1* knockdown and control HAoECs. The graph represents percentage apoptotic ECs calculated by dividing the number of active caspase-3-positive cells by the total number of EC per field of view (*n* = 4 donors). (*D* and *E*) HAoECs were treated with *PKD1* siRNA or with NTC siRNA and cultured on Transwell inserts for 72 h following transfection. EC permeability was assessed after adding Rd-albumin as a tracer to the top compartment for 1 h. The graph in (*E*) represents concentration of Rd-albumin measured in the lower compartment (*n* = 3 donors). Differences between groups were analysed using non-parametric Mann–Whitney test (*A*) or unpaired *t*-test (*B* and *E*), with *P* values shown in the graphs. Scale bar: (*C*) 50 µm.

Increased EC turnover due to elevated apoptosis levels can result in increased permeability of the EC monolayer, which is relevant for cardiovascular disease development.^69^ We therefore assessed the effect of *PKD1* knockdown on EC permeability using the Transwell assay (*Figure 4D* and *E*). Following 72 h of transfection, *PKD1*-deficient HAoECs were more permeable to fluorescent tracer, Rd-labelled albumin (37.58 ± 0.38 µg/mL) compared with control transfected cells (5.29 ± 1.82 µg/mL, *P* < 0.0001). These results indicate that, in agreement with our observations in zebrafish embryos and mouse aortas, PKD1 promotes survival of human ECs and, additionally, that PKD1 regulates endothelial barrier function by inhibiting endothelial permeability.

To gain further mechanistic insights, we tested whether THBS1 and CCN1 are downstream targets of PKD1 in human ECs. Knockdown of *PKD1* resulted in a significant increase of mRNA expression of both *THBS1* (2.25-fold, *P* = 0.0286) and *CCN1* (3.04-fold, *P* = 0.0286) (*Figure 5A* and *B*). Concomitantly, we detected decreased expression of *NOS3* gene, which encodes for eNOS, in *PKD1*-deficient ECs compared with control-transfected cells (0.21-fold, *P* = 0.0286) (*Figure 5C*), as well as reduced NO production (see Supplementary material online, Figure  *S15A*). Consistent trends were observed under defined flow conditions, although knockdown efficiency was reduced (see Supplementary material online, Figure  *S16*).

**Figure 5:**
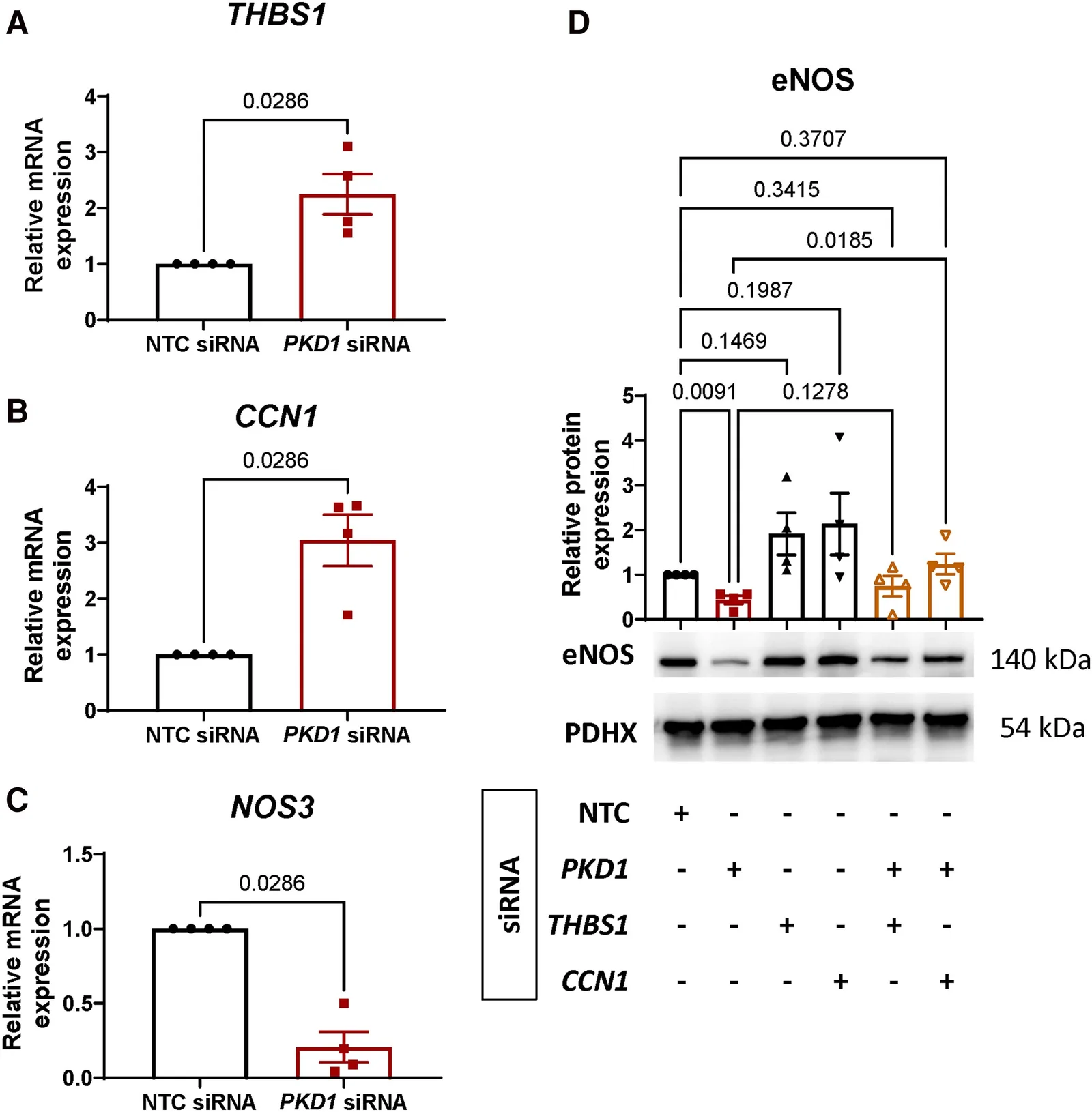
THBS1, CCN1, and eNOS are downstream targets of PKD1 in HAoEC. (*A-C*) HAoECs were treated with 10 nM *PKD1* siRNA or with NTC siRNA for 72 h. Expression of *THBS1*, *CCN1*, and *NOS3* was measured by qPCR (*n* = 4 donors) using *HPRT* as a housekeeping gene. (*D*) HAoECs were treated with *PKD1* siRNA, *THBS1* siRNA, and/or *CCN1* siRNA for 72 h, as indicated in the image. Control cells were treated with NTC siRNA. Expression levels of eNOS were assessed by western blotting. PDHX was used as a loading control. Graph shows relative protein levels of eNOS normalized to PDHX (*n* = 4 donors). Differences between groups were analysed using non-parametric Mann–Whitney test (*A–C*) or one-way ANOVA with Sidak's multiple comparisons *post-hoc* test (*D*), with *P* values shown in the graphs.

Based on these findings, we hypothesized that induction of *THBS1* and *CCN1* in the absence of PKD1 mediates the inhibition of eNOS. We therefore tested whether knockdown of *THBS1* or *CCN1* in *PKD1*-deficient cells can rescue decreased eNOS expression caused by *PKD1* knockdown (*Figure 5D*). Knockdown of *PKD1* led to decreased eNOS protein levels (0.44-fold, *P* = 0.0091). Transfection of HAoECs with gene-specific siRNAs targeting *THBS1* or *CCN1* resulted in decreased mRNA expression of *THBS1* and *CCN1* by 84 and 64%, respectively (see Supplementary material online, Figure  *S15B–C*). Silencing of *THBS1* in *PKD1*-deficient cells resulted in a trend towards rescued eNOS expression compared with *PKD1*-deficient cells (0.74-fold vs. control, *P* = 0.3415), (*Figure 5D*). On the other hand, silencing of *CCN1* in *PKD1*-deficient cells resulted in restored expression of eNOS to levels comparable to control-transfected cells (1.24-fold vs. control, *P* = 0.3707), (*Figure 5D*).

We next tested whether down-regulation of THSB1 and CCN1 by PKD1 mediates the effect of PKD1 on endothelial survival. Knockdown of *PKD1* significantly increased EC apoptosis (1.80 ± 0.15% apoptotic ECs, *P* = 0.0038 vs. control) compared with control cells (0.72 ± 0.19% apoptotic ECs), which was restored to apoptosis levels comparable to control cells by silencing *THBS1* (1.02 ± 0.11% apoptotic ECs, *P* = 0.3334 vs. control) or *CCN1* (0.99 ± 0.34% apoptotic ECs, *P* = 0.3811 vs. control) in *PKD1*-deficient cells (*Figure 6A* and *B*). LDH assay of media collected from the transfected cells 3 days after transfection indicated increased cell death of *PKD1*-deficient ECs compared with control cells by approximately 2.7-fold, which was rescued by knocking down *THBS1* (0.85-fold vs. control) or *CCN1* (0.88-fold vs. control) in *PKD1*-silenced cells (see Supplementary material online, Figure  *S15D*).

**Figure 6:**
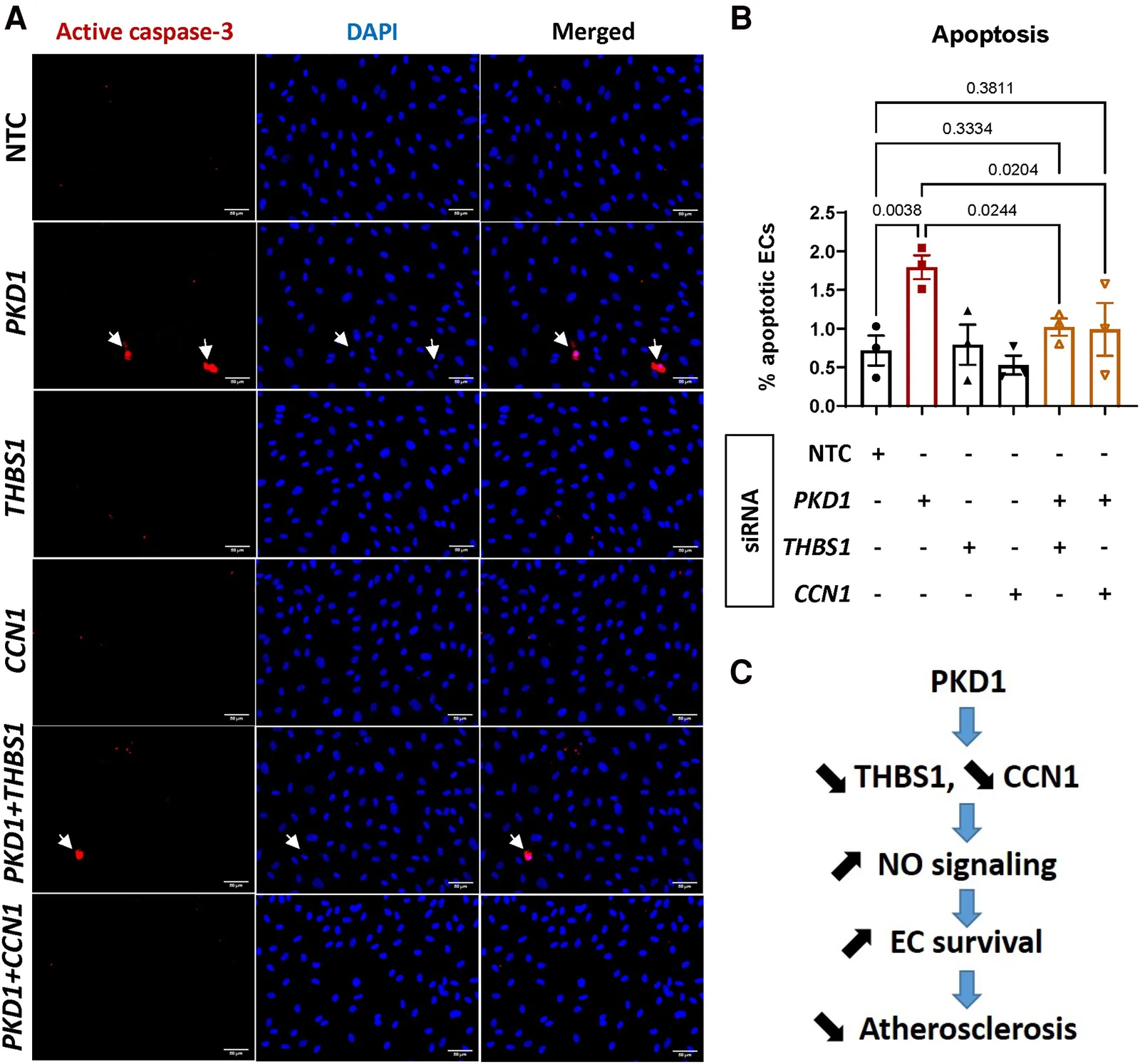
PKD1 protects from endothelial apoptosis by inhibiting THBS1 and CCN1. (*A* and *B*) HAoECs were treated with *PKD1* siRNA, *THBS1* siRNA, and/or *CCN1* siRNA for 72 h, as indicated in the images. Control cells were treated with NTC siRNA. (*A*) EC apoptosis was assessed by immunostaining using anti-active caspase-3 antibody and DAPI. Apoptotic HAoECs are indicated by arrows. (*B*) Quantification of EC apoptosis. The graph represents percentage apoptotic ECs calculated by dividing the number of active caspase-3-positive cells by the total number of EC per field of view (*n* = 3 donors). (*C*) A schematic diagram summarising proposed mechanism how PKD1 regulates endothelial survival and protects from atherosclerosis development. PKD1 contributes to increased EC survival by inhibiting expression of THBS1 and CCN1 and promoting NO signalling, resulting in athero-protection. Differences between groups were analysed using one-way ANOVA with Tukey's *post-hoc* test (*B*), with *P* values shown in the graph. Scale bar: (*A*) 50 µm.

The association of THBS1 and CCN1 with TGFβ pathway and EndMT, prompted us to examine expression of EC and mesenchymal markers in *PKD1*-silenced HAoEC (see Supplementary material online, Figure  *S17*). Although EC markers (*CDH5, PECAM1*) were decreased, there was no consistent change in mesenchymal markers (*ACTA2, CDH2, CTGF*), indicating that the *PKD1*-deficient ECs may be undergoing partial EndMT.

Finally, to provide translational relevance to our findings, we used the Kidney Interactive Transcriptomics platform (https://humphreyslab.com/SingleCell/) to analyse a publicly available scRNAseq dataset of control and ADPKD human patient kidneys.^54^ Within the EC cluster, we found that THBS1 and CCN1 were highly up-regulated in ADKPD patient kidneys compared with control kidneys (see Supplementary material online, Figure  *S18*). This up-regulation was, interestingly, not specific to ECs and was observed in other cell clusters from the kidney, indicating that PKD1-regulated pathways are conserved across different cell types, including ECs (see Supplementary material online, Figure  *S18*).

Taken together, these data demonstrate that PKD1 exerts its protective effect in the endothelium, at least in part, by inhibiting expression of THBS1 and CCN1 and up-regulating downstream NO signalling (*Figure 6C*).

## 4. Discussion

Endothelial apoptosis plays a key role in the pathogenesis of atherosclerosis, by enhancing permeability of the arterial wall to cells and cholesterol-rich lipoproteins in athero-prone regions of arteries.^69–71^ This focal nature of atherosclerotic lesions is determined by responses of ECs to local haemodynamic forces via mechanoreceptors on their surface. There is, however, a paucity of knowledge of how blood flow-induced forces link to downstream signalling pathways and EC function.

In this study, we used zebrafish embryos to screen a range of known and putative EC mechanoreceptors for their ability to protect ECs from apoptosis. The zebrafish has several advantages for analysis of gene function *in vivo* as it is small, easily maintained, develops rapidly and can be genetically manipulated, making it suitable for screening studies of gene function. It has proved to be a useful model for studying cardiovascular development and modelling cardiovascular diseases^72^ and our groups and others have demonstrated that many of the endothelial responses to flow are conserved in zebrafish.^47,73–76^ Our functional study identified two polycystin proteins (PKD1 and PKD2) as protective factors in the zebrafish endothelium in a flow-dependent manner.

PKD1 and PKD2 are required for normal embryonic development and mice lacking either of the two genes exhibit oedema, vascular leaks, and rupture of blood vessels, indicating their important role in maintaining structural integrity of the vasculature.^77,78^ Both molecules have been described to play diverse functions in a range of cell types in addition to ECs, including kidney epithelial cells, VSMCs, cardiomyocytes and bone osteoblasts.^11^ Therefore, to specifically assess the role of endothelial PKD1 and PKD2 in atherosclerosis, it was required to generate mouse lines with an EC-specific deletion of *Pkd1* or *Pkd2*. Interestingly, endothelial PKD1, but not PKD2, had a protective effect on atherosclerosis development. Additionally, PKD1, but not PKD2, was required for promoting EC survival of HAoEC. This suggests that PKD1 may function as the dominant regulator of endothelial survival in mammalian systems, whereas PKD2 may play a more context-dependent or developmental role. PKD1 and PKD2 can form a complex^14,79^ and work together to regulate diverse cellular functions, however they can also play separate, and even opposing roles in different cellular contexts.^80^ Hence, PKD1 may be involved in atherosclerosis development independently of PKD2, or alternatively, PKD1 may be partially compensating for PKD2 deficiency in *Pkd2^iECKO^* mice. Further studies are required to dissect potential role of PKD2 in protective effects in ECs and atherosclerosis development.

Our transcriptomic analysis of *Pkd1*-deficient and control mouse aortic ECs identified a number of potential downstream targets, which could be mediating the athero-protective effect of PKD1. We were able to validate in mouse and human aortic ECs that expression of two matricellular proteins, THBS1 and CCN1, is negatively regulated by PKD1. Both molecules are known to promote EC apoptosis^57,61^ have a pro-atherogenic effect,^58,61^ and have been linked to PKD1-implicated pathways such as TGFβ and YAP/TAZ in other cellular contexts,^81–84^ making them good candidates for PKD1 downstream targets in the endothelium. THBS1 and CCN1 are recognized as markers and drivers of EndMT, particularly under pathological conditions, such as atherosclerosis. During EndMT, ECs lose endothelial and obtain mesenchymal phenotype, become migratory and can contribute to atherosclerotic plaque development.^85^ Our data indicates that *PKD1* silencing induces modest changes in mesenchymal gene expression *in vitro*, although further studies are required to determine whether this reflects functional EndoMT *in vivo*. Additionally, both THBS1^59,60^ and CCN1^61^ have been linked to eNOS and NO signalling. The small molecule NO, synthesized by the shear-sensitive, endothelial-specific enzyme eNOS, is an important protective factor in the cardiovascular system, playing multiple roles including vasodilation and anti-thrombotic, anti-inflammatory and anti-apoptotic functions.^86,87^ ADPKD patients exhibit a loss of NO release and an associated reduction in endothelium-dependent dilatation of conduit arteries at an early stage of the disease.^88^ Moreover, recent studies have shown that endothelial-specific deletion of *Pkd1* in mice attenuates flow-mediated vasodilation and leads to arterial hypertension, independently of alterations in kidney function and structure.^29,30^ Our study builds on these findings and identifies THBS1 and CCN1 as novel downstream targets of PKD1. Although our data across zebrafish, murine and human endothelial systems consistently support a flow-dependent role for PKD1, direct mechanistic interrogation under defined flow conditions in mammalian ECs remains technically challenging. Notably, *in vitro* experiments under shear stress conditions demonstrated consistent directional effects on PKD1-regulated pathways despite reduced knockdown efficiency, supporting a role for PKD1 in endothelial responses to haemodynamic forces.

PKD1, localized to cilia and cell membrane, has been suggested to act as mechanoreceptor of urine flow,^13^ blood flow^89^ and cerebrospinal fluid.^90^ In mouse ECs, PKD1 was shown to be required for wall shear stress–dependent changes in calcium and NO.^89^ However, it remains unknown whether PKD1 is indeed a direct sensor of mechanical force in ECs. This can be addressed by the use of magnetic tweezers to test whether localized mechanical stimulation of PKD1 can lead to a cellular response, as was previously shown for PECAM1,^3^ PLXND1,^6^ and ITGB1.^9^ We propose that DF-induced up-regulation of PKD1 represents a protective adaptive response, analogous to other mechanosensitive pathways, including primary cilia. Endothelial primary cilia, which are more common in the athero-prone regions of arteries, inhibit atherosclerosis^91^ and this protective effect may be in part mediated by PKD1. Dissecting the role of cell membrane- and cilia-associated PKD1 will be an important subject of future studies.

In summary, by integrating zebrafish, mouse and human models for studying EC function with single cell transcriptomics, we have identified a new role for PKD1 in regulating endothelial survival and inhibiting atherosclerotic lesion development, and have provided mechanistic insights into PKD1-mediated protective signalling pathways.

## Supplementary Material

cvag171_Supplementary_Data

## Data Availability

The data underlying this article will be shared on reasonable request to the corresponding author.

## References

[1] Brown AJ, Teng Z, Evans PC, Gillard JH, Samady H, Bennett MR. Role of biomechanical forces in the natural history of coronary atherosclerosis. Nat Rev Cardiol 2016;13:210–220. 10.1038/nrcardio.2015.203 26822720

[2] Kwak BR, Back M, Bochaton-Piallat ML, Caligiuri G, Daemen MJ, Davies PF, Hoefer IE, Holvoet P, Jo H, Krams R, Lehoux S, Monaco C, Steffens S, Virmani R, Weber C, Wentzel JJ, Evans PC. Biomechanical factors in atherosclerosis: mechanisms and clinical implications. Eur Heart J 2014;35:3013–3020d. 10.1093/eurheartj/ehu353 PMC4810806 25230814

[3] Tzima E, Irani-Tehrani M, Kiosses WB, Dejana E, Schultz DA, Engelhardt B, Cao G, DeLisser H, Schwartz MA. A mechanosensory complex that mediates the endothelial cell response to fluid shear stress. Nature 2005;437:426–431. 10.1038/nature03952 16163360

[4] Ranade SS, Qiu ZZ, Woo SH, Hur SS, Murthy SE, Cahalan SM, Xu J, Mathur J, Bandell M, Coste B, Li YSJ, Chien S, Patapoutian A. Piezo1, a mechanically activated ion channel, is required for vascular development in mice. Proc Natl Acad Sci U S A 2014;111:10347–10352. 10.1073/pnas.1409233111 PMC4104881 24958852

[5] Li J, Hou B, Tumova S, Muraki K, Bruns A, Ludlow MJ, Sedo A, Hyman AJ, McKeown L, Young RS, Yuldasheva NY, Majeed Y, Wilson LA, Rode B, Bailey MA, Kim HR, Fu Z, Carter DA, Bilton J, Imrie H, Ajuh P, Dear TN, Cubbon RM, Kearney MT, Prasad RK, Evans PC, Ainscough JF, Beech DJ. Piezo1 integration of vascular architecture with physiological force. Nature 2014;515:279–282. 10.1038/nature13701 PMC4230887 25119035

[6] Mehta V, Pang KL, Rozbesky D, Nather K, Keen A, Lachowski D, Kong Y, Karia D, Ameismeier M, Huang J, Fang Y, Del Rio Hernandez A, Reader JS, Jones EY, Tzima E. The guidance receptor plexin D1 is a mechanosensor in endothelial cells. Nature 2020;578:290–295. 10.1038/s41586-020-1979-4 PMC7025890 32025034

[7] Givens C, Tzima E. Endothelial mechanosignaling: does one sensor fit all? Antioxid Redox Signal 2016;25:373–388. 10.1089/ars.2015.6493 PMC5011625 27027326

[8] Chuntharpursat-Bon E, Povstyan OV, Ludlow MJ, Carrier DJ, Debant M, Shi J, Gaunt HJ, Bauer CC, Curd A, Simon Futers T, Baxter PD, Peckham M, Muench SP, Adamson A, Humphreys N, Tumova S, Bon RS, Cubbon R, Lichtenstein L, Beech DJ. PIEZO1 and PECAM1 interact at cell-cell junctions and partner in endothelial force sensing. Commun Biol 2023;6:358. 10.1038/s42003-023-04706-4 PMC10067937 37005489

[9] Xanthis I, Souilhol C, Serbanovic-Canic J, Roddie H, Kalli AC, Fragiadaki M, Wong R, Shah DR, Askari JA, Canham L, Akhtar N, Feng S, Ridger V, Waltho J, Pinteaux E, Humphries MJ, Bryan MT, Evans PC. Beta1 integrin is a sensor of blood flow direction. J Cell Sci 2019;132:. 10.1242/jcs.229542 PMC6589088 31076511

[10] Souilhol C, Serbanovic-Canic J, Fragiadaki M, Chico TJ, Ridger V, Roddie H, Evans PC. Endothelial responses to shear stress in atherosclerosis: a novel role for developmental genes. Nat Rev Cardiol 2020;17:52–63. 10.1038/s41569-019-0239-5 31366922

[11] Nigro EA, Boletta A. Role of the polycystins as mechanosensors of extracellular stiffness. Am J Physiol Renal Physiol 2021;320:F693–F705. 10.1152/ajprenal.00545.2020 33615892

[12] AbouAlaiwi WA, Takahashi M, Mell BR, Jones TJ, Ratnam S, Kolb RJ, Nauli SM. Ciliary polycystin-2 is a mechanosensitive calcium channel involved in nitric oxide signaling cascades. Circ Res 2009;104:860–869. 10.1161/CIRCRESAHA.108.192765 PMC3085025 19265036

[13] Nauli SM, Alenghat FJ, Luo Y, Williams E, Vassilev P, Li X, Elia AE, Lu W, Brown EM, Quinn SJ, Ingber DE, Zhou J. Polycystins 1 and 2 mediate mechanosensation in the primary cilium of kidney cells. Nat Genet 2003;33:129–137. 10.1038/ng1076 12514735

[14] Su Q, Hu F, Ge X, Lei J, Yu S, Wang T, Zhou Q, Mei C, Shi Y. Structure of the human PKD1-PKD2 complex. Science 2018;361:eaat9819. 10.1126/science.aat9819 30093605

[15] Torres VE, Harris PC, Pirson Y. Autosomal dominant polycystic kidney disease. Lancet 2007;369:1287–1301. 10.1016/S0140-6736(07)60601-1 17434405

[16] Vasileva VY, Sultanova RF, Sudarikova AV, Ilatovskaya DV. Insights into the molecular mechanisms of polycystic kidney diseases. Front Physiol 2021;12:693130. 10.3389/fphys.2021.693130 PMC8456103 34566674

[17] Nowak KL, Edelstein CL. Apoptosis and autophagy in polycystic kidney disease (PKD). Cell Signal 2020;68:109518. 10.1016/j.cellsig.2019.109518 PMC7127985 31881325

[18] Lemos FO, Ehrlich BE. Polycystin and calcium signaling in cell death and survival. Cell Calcium 2018;69:37–45. 10.1016/j.ceca.2017.05.011 PMC5701862 28601384

[19] Bhunia AK, Piontek K, Boletta A, Liu L, Qian F, Xu PN, Germino FJ, Germino GG. PKD1 induces p21(waf1) and regulation of the cell cycle via direct activation of the JAK-STAT signaling pathway in a process requiring PKD2. Cell 2002;109:157–168. 10.1016/s0092-8674(02)00716-x 12007403

[20] Song X, Di Giovanni V, He N, Wang K, Ingram A, Rosenblum ND, Pei Y. Systems biology of autosomal dominant polycystic kidney disease (ADPKD): computational identification of gene expression pathways and integrated regulatory networks. Hum Mol Genet 2009;18:2328–2343. 10.1093/hmg/ddp165 19346236

[21] Torres VE, Harris PC. Strategies targeting cAMP signaling in the treatment of polycystic kidney disease. J Am Soc Nephrol 2014;25:18–32. 10.1681/ASN.2013040398 PMC3871779 24335972

[22] Qiu J, Germino GG, Menezes LF. Mechanisms of cyst development in polycystic kidney disease. Adv Kidney Dis Health 2023;30:209–219. 10.1053/j.akdh.2023.03.001 PMC10289784 37088523

[23] Ecder T, Schrier RW. Cardiovascular abnormalities in autosomal-dominant polycystic kidney disease. Nat Rev Nephrol 2009;5:221–228. 10.1038/nrneph.2009.13 PMC2720315 19322187

[24] Kuo IY, Chapman AB. Polycystins, ADPKD, and cardiovascular disease. Kidney Int Rep 2020;5:396–406. 10.1016/j.ekir.2019.12.007 PMC7136326 32274448

[25] Perrone RD, Ruthazer R, Terrin NC. Survival after end-stage renal disease in autosomal dominant polycystic kidney disease: contribution of extrarenal complications to mortality. Am J Kidney Dis 2001;38:777–784. 10.1053/ajkd.2001.27720 11576881

[26] Kocaman O, Oflaz H, Yekeler E, Dursun M, Erdogan D, Demirel S, Alisir S, Turgut F, Mercanoglu F, Ecder T. Endothelial dysfunction and increased carotid intima-media thickness in patients with autosomal dominant polycystic kidney disease. Am J Kidney Dis 2004;43:854–860. 10.1053/j.ajkd.2004.01.011 15112176

[27] Turkmen K, Oflaz H, Uslu B, Cimen AO, Elitok A, Kasikcioglu E, Alisir S, Tufan F, Namli S, Uysal M, Ecder T. Coronary flow velocity reserve and carotid intima media thickness in patients with autosomal dominant polycystic kidney disease: from impaired tubules to impaired carotid and coronary arteries. Clin J Am Soc Nephrol 2008;3:986–991. 10.2215/CJN.02330607 PMC2440264 18354076

[28] MacKay CE, Leo MD, Fernandez-Pena C, Hasan R, Yin W, Mata-Daboin A, Bulley S, Gammons J, Mancarella S, Jaggar JH. Intravascular flow stimulates PKD2 (polycystin-2) channels in endothelial cells to reduce blood pressure. Elife 2020;9:e56655. 10.7554/eLife.56655 PMC7228764 32364494

[29] MacKay CE, Floen M, Leo MD, Hasan R, Garrud TAC, Fernandez-Pena C, Singh P, Malik KU, Jaggar JH. A plasma membrane-localized polycystin-1/polycystin-2 complex in endothelial cells elicits vasodilation. Elife 2022;11:e74765. 10.7554/eLife.74765 PMC8933003 35229718

[30] Hamzaoui M, Groussard D, Nezam D, Djerada Z, Lamy G, Tardif V, Dumesnil A, Renet S, Brunel V, Peters DJM, Chevalier L, Hanoy M, Mulder P, Richard V, Bellien J, Guerrot D. Endothelium-specific deficiency of polycystin-1 promotes hypertension and cardiovascular disorders. Hypertension 2022;79:2542–2551. 10.1161/HYPERTENSIONAHA.122.19057 36093769

[31] Albarran-Juarez J, Iring A, Wang S, Joseph S, Grimm M, Strilic B, Wettschureck N, Althoff TF, Offermanns S. Piezo1 and Gq/G11 promote endothelial inflammation depending on flow pattern and integrin activation. J Exp Med 2018;215:2655–2672. 10.1084/jem.20180483 PMC6170174 30194266

[32] Westerfield M . The Zebrafish Book: A Guide for the Laboratory Use of Zebrafish (Danio Rerio). 5th Edition. Eugene: University of Oregon Press; 2007.

[33] Kimmel CB, Ballard WW, Kimmel SR, Ullmann B, Schilling TF. Stages of embryonic development of the zebrafish. Dev Dyn 1995;203:253–310. 10.1002/aja.1002030302 8589427

[34] Covassin L, Amigo JD, Suzuki K, Teplyuk V, Straubhaar J, Lawson ND. Global analysis of hematopoietic and vascular endothelial gene expression by tissue specific microarray profiling in zebrafish. Dev Biol 2006;299:551–562. 10.1016/j.ydbio.2006.08.020 PMC1779954 16999953

[35] Thisse C, Thisse B. High-resolution in situ hybridization to whole-mount zebrafish embryos. Nat Protoc 2008;3:59–69. 10.1038/nprot.2007.514 18193022

[36] Gray C, Bratt D, Lees J, daCosta M, Plant K, Watson OJ, Solaymani-Kohal S, Tazzyman S, Serbanovic-Canic J, Crossman DC, Keavney BD, Haase A, McMahon K, Gering M, Roehl H, Evans PC, Chico TJ. Loss of function of parathyroid hormone receptor 1 induces Notch-dependent aortic defects during zebrafish vascular development. Arterioscler Thromb Vasc Biol 2013;33:1257–1263. 10.1161/ATVBAHA.112.300590 23559631

[37] Piontek KB, Huso DL, Grinberg A, Liu L, Bedja D, Zhao H, Gabrielson K, Qian F, Mei C, Westphal H, Germino GG. A functional floxed allele of Pkd1 that can be conditionally inactivated in vivo. J Am Soc Nephrol 2004;15:3035–3043. 10.1097/01.ASN.0000144204.01352.86 15579506

[38] Garcia-Gonzalez MA, Outeda P, Zhou Q, Zhou F, Menezes LF, Qian F, Huso DL, Germino GG, Piontek KB, Watnick T. Pkd1 and Pkd2 are required for normal placental development. PLoS One 2010;5:e12821. 10.1371/journal.pone.0012821 PMC2940908 20862291

[39] Wang Y, Nakayama M, Pitulescu ME, Schmidt TS, Bochenek ML, Sakakibara A, Adams S, Davy A, Deutsch U, Luthi U, Barberis A, Benjamin LE, Makinen T, Nobes CD, Adams RH. Ephrin-B2 controls VEGF-induced angiogenesis and lymphangiogenesis. Nature 2010;465:483–486. 10.1038/nature09002 20445537

[40] Bjorklund MM, Hollensen AK, Hagensen MK, Dagnaes-Hansen F, Christoffersen C, Mikkelsen JG, Bentzon JF. Induction of atherosclerosis in mice and hamsters without germline genetic engineering. Circ Res 2014;114:1684–1689. 10.1161/CIRCRESAHA.114.302937 24677271

[41] Nam D, Ni CW, Rezvan A, Suo J, Budzyn K, Llanos A, Harrison D, Giddens D, Jo H. Partial carotid ligation is a model of acutely induced disturbed flow, leading to rapid endothelial dysfunction and atherosclerosis. Am J Physiol Heart Circ Physiol 2009;297:H1535–H1543. 10.1152/ajpheart.00510.2009 PMC2770764 19684185

[42] Dunn J, Qiu H, Kim S, Jjingo D, Hoffman R, Kim CW, Jang I, Son DJ, Kim D, Pan C, Fan Y, Jordan IK, Jo H. Flow-dependent epigenetic DNA methylation regulates endothelial gene expression and atherosclerosis. J Clin Invest 2014;124:3187–3199. 10.1172/JCI74792 PMC4071393 24865430

[43] Muraro MJ, Dharmadhikari G, Grun D, Groen N, Dielen T, Jansen E, van Gurp L, Engelse MA, Carlotti F, de Koning EJ, van Oudenaarden A. A single-cell transcriptome atlas of the human pancreas. Cell Syst 2016;3:385–394 e383. 10.1016/j.cels.2016.09.002 PMC5092539 27693023

[44] Hashimshony T, Senderovich N, Avital G, Klochendler A, de Leeuw Y, Anavy L, Gennert D, Li S, Livak KJ, Rozenblatt-Rosen O, Dor Y, Regev A, Yanai I. CEL-Seq2: sensitive highly-multiplexed single-cell RNA-Seq. Genome Biol 2016;17:77. 10.1186/s13059-016-0938-8 PMC4848782 27121950

[45] Souilhol C, Tardajos Ayllon B, Li X, Diagbouga MR, Zhou Z, Canham L, Roddie H, Pirri D, Chambers EV, Dunning MJ, Ariaans M, Li J, Fang Y, Jorgensen HF, Simons M, Krams R, Waltenberger J, Fragiadaki M, Ridger V, De Val S, Francis SE, Chico TJ, Serbanovic-Canic J, Evans PC. JAG1-NOTCH4 mechanosensing drives atherosclerosis. Sci Adv 2022;8:eabo7958. 10.1126/sciadv.abo7958 PMC9432841 36044575

[46] Warboys CM, Berson E, Mann R, Pearson GE, Weinberg JD, D P. Acute and chronic exposure to shear stress have opposite effects on endothelial permeability to macromolecules. Am J Physiol Heart Circ Physiol 2010;298:H1850–H1856. 10.1152/ajpheart.00114.2010 PMC2886649 20363882

[47] Serbanovic-Canic J, de Luca A, Warboys C, Ferreira PF, Luong LA, Hsiao S, Gauci I, Mahmoud M, Feng S, Souilhol C, Bowden N, Ashton JP, Walczak H, Firmin D, Krams R, Mason JC, Haskard DO, Sherwin S, Ridger V, Chico TJ, Evans PC. Zebrafish model for functional screening of flow-responsive genes. Arterioscler Thromb Vasc Biol 2017;37:130–143. 10.1161/ATVBAHA.116.308502 PMC5172514 27834691

[48] Fang Y, Wu D, Birukov KG. Mechanosensing and mechanoregulation of endothelial cell functions. Compr Physiol 2019;9:873–904. 10.1002/cphy.c180020 PMC6697421 30873580

[49] Faucherre A, Kissa K, Nargeot J, Mangoni ME, Jopling C. Piezo1 plays a role in erythrocyte volume homeostasis. Haematologica 2014;99:70–75. 10.3324/haematol.2013.086090 PMC4007942 23872304

[50] Sun Z, Amsterdam A, Pazour GJ, Cole DG, Miller MS, Hopkins N. A genetic screen in zebrafish identifies cilia genes as a principal cause of cystic kidney. Development 2004;131:4085–4093. 10.1242/dev.01240 15269167

[51] Schottenfeld J, Sullivan-Brown J, Burdine RD. Zebrafish curly up encodes a Pkd2 ortholog that restricts left-side-specific expression of southpaw. Development 2007;134:1605–1615. 10.1242/dev.02827 17360770

[52] Albarran-Juarez J, Iring A, Wang S, Joseph S, Grimm M, Strilic B, Wettschureck N, Althoff TF, Offermanns S. Piezo1 and G(q)/G(11) promote endothelial inflammation depending on flow pattern and integrin activation. J Exp Med 2018;215:2655–2672. 10.1084/jem.20180483 PMC6170174 30194266

[53] Beech DJ, Kalli AC. Force sensing by piezo channels in cardiovascular health and disease. Arterioscler Thromb Vasc Biol 2019;39:2228–2239. 10.1161/ATVBAHA.119.313348 PMC6818984 31533470

[54] Muto Y, Dixon EE, Yoshimura Y, Wu H, Omachi K, Ledru N, Wilson PC, King AJ, Olson E, Gunawan N, Kuo MG, Cox JJ, Miner JH, Seliger JH, Woodward SL, Welling OM, Watnick PA, Humphreys TJ, D B. Defining cellular complexity in human autosomal dominant polycystic kidney disease by multimodal single cell analysis. Nat Commun 2022;13:6497. 10.1038/s41467-022-34255-z PMC9618568 36310237

[55] Kalluri AS, Vellarikkal SK, Edelman ER, Nguyen L, Subramanian A, Ellinor PT, Regev A, Kathiresan S, Gupta RM. Single-cell analysis of the normal mouse aorta reveals functionally distinct endothelial cell populations. Circulation 2019;140:147–163. 10.1161/CIRCULATIONAHA.118.038362 PMC6693656 31146585

[56] Engelbrecht E, Levesque MV, He L, Vanlandewijck M, Nitzsche A, Niazi H, Kuo A, Singh SA, Aikawa M, Holton K, Proia RL, Kono M, Pu WT, Camerer E, Betsholtz C, Hla T. Sphingosine 1-phosphate-regulated transcriptomes in heterogenous arterial and lymphatic endothelium of the aorta. Elife 2020;9:e52690. 10.7554/eLife.52690 PMC7054001 32091396

[57] Nor JE, Mitra RS, Sutorik MM, Mooney DJ, Castle VP, Polverini PJ. Thrombospondin-1 induces endothelial cell apoptosis and inhibits angiogenesis by activating the caspase death pathway. J Vasc Res 2000;37:209–218. 10.1159/000025733 10859480

[58] Moura R, Tjwa M, Vandervoort P, Cludts K, Hoylaerts MF. Thrombospondin-1 activates medial smooth muscle cells and triggers neointima formation upon mouse carotid artery ligation. Arterioscler Thromb Vasc Biol 2007;27:2163–2169. 10.1161/ATVBAHA.107.151282 17761938

[59] Nevitt C, McKenzie G, Christian K, Austin J, Hencke S, Hoying J, LeBlanc A. Physiological levels of thrombospondin-1 decrease NO-dependent vasodilation in coronary microvessels from aged rats. Am J Physiol Heart Circ Physiol 2016;310:H1842–H1850. 10.1152/ajpheart.00086.2016 PMC6345213 27199114

[60] Bauer EM, Qin Y, Miller TW, Bandle RW, Csanyi G, Pagano PJ, Bauer PM, Schnermann J, Roberts DD, Isenberg JS. Thrombospondin-1 supports blood pressure by limiting eNOS activation and endothelial-dependent vasorelaxation. Cardiovasc Res 2010;88:471–481. 10.1093/cvr/cvq218 PMC2972685 20610415

[61] Hsu PL, Chen JS, Wang CY, Wu HL, Mo FE. Shear-induced CCN1 promotes atheroprone endothelial phenotypes and atherosclerosis. Circulation 2019;139:2877–2891. 10.1161/CIRCULATIONAHA.118.033895 30917686

[62] Tian XY, Yung LH, Wong WT, Liu J, Leung FP, Liu L, Chen Y, Kong SK, Kwan KM, Ng SM, Lai PB, Yung LM, Yao X, Huang Y. Bone morphogenic protein-4 induces endothelial cell apoptosis through oxidative stress-dependent p38MAPK and JNK pathway. J Mol Cell Cardiol 2012;52:237–244. 10.1016/j.yjmcc.2011.10.013 22064324

[63] Sorescu GP, Sykes M, Weiss D, Platt MO, Saha A, Hwang J, Boyd N, Boo YC, Vega JD, Taylor WR, Jo H. Bone morphogenic protein 4 produced in endothelial cells by oscillatory shear stress stimulates an inflammatory response. J Biol Chem 2003;278:31128–31135. 10.1074/jbc.M300703200 12766166

[64] Koga M, Yamauchi A, Kanaoka Y, Jige R, Tsukamoto A, Teshima N, Nishioku T, Kataoka Y. BMP4 is increased in the aortas of diabetic ApoE knockout mice and enhances uptake of oxidized low density lipoprotein into peritoneal macrophages. J Inflamm (Lond) 2013;10:32. 10.1186/1476-9255-10-32 PMC3852969 24107300

[65] Youn JY, Zhou J, Cai H. Bone morphogenic protein 4 mediates NOX1-dependent eNOS uncoupling, endothelial dysfunction, and COX2 induction in type 2 diabetes mellitus. Mol Endocrinol 2015;29:1123–1133. 10.1210/ME.2014-1313 PMC4518001 26121233

[66] Sullivan DC, Huminiecki L, Moore JW, Boyle JJ, Poulsom R, Creamer D, Barker J, Bicknell R. EndoPDI, a novel protein-disulfide isomerase-like protein that is preferentially expressed in endothelial cells acts as a stress survival factor. J Biol Chem 2003;278:47079–47088. 10.1074/jbc.M308124200 12963716

[67] Camargo LL, Babelova A, Mieth A, Weigert A, Mooz J, Rajalingam K, Heide H, Wittig I, Lopes LR, Brandes RP. Endo-PDI is required for TNFalpha-induced angiogenesis. Free Radic Biol Med 2013;65:1398–1407. 10.1016/j.freeradbiomed.2013.09.028 24103565

[68] Yeh CF, Cheng SH, Lin YS, Shentu TP, Huang RT, Zhu J, Chen YT, Kumar S, Lin MS, Kao HL, Huang PH, Rosello-Sastre E, Garcia F, Jo H, Fang Y, Yang KC. Targeting mechanosensitive endothelial TXNDC5 to stabilize eNOS and reduce atherosclerosis in vivo. Sci Adv 2022;8:eabl8096. 10.1126/sciadv.abl8096 PMC8782452 35061532

[69] Mundi S, Massaro M, Scoditti E, Carluccio MA, van Hinsbergh VWM, Iruela-Arispe ML, De Caterina R. Endothelial permeability, LDL deposition, and cardiovascular risk factors-a review. Cardiovasc Res 2018;114:35–52. 10.1093/cvr/cvx226 PMC7729208 29228169

[70] Foteinos G, Hu Y, Xiao Q, Metzler B, Xu Q. Rapid endothelial turnover in atherosclerosis-prone areas coincides with stem cell repair in apolipoprotein E-deficient mice. Circulation 2008;117:1856–1863. 10.1161/CIRCULATIONAHA.107.746008 18378610

[71] Stoneman VE, Bennett MR. Role of apoptosis in atherosclerosis and its therapeutic implications. Clin Sci (Lond) 2004;107:343–354. 10.1042/CS20040086 15230690

[72] Bowley G, Kugler E, Wilkinson R, Lawrie A, van Eeden F, Chico TJA, Evans PC, Noel ES, Serbanovic-Canic J. Zebrafish as a tractable model of human cardiovascular disease. Br J Pharmacol 2022;179:900–917. 10.1111/bph.15473 33788282

[73] Canham L, Sendac S, Diagbouga MR, Wolodimeroff E, Pirri D, Tardajos Ayllon B, Feng S, Souilhol C, Chico TJA, Evans PC, Serbanovic-Canic J. EVA1A (Eva-1 Homolog A) promotes endothelial apoptosis and inflammatory activation under disturbed flow via regulation of autophagy. Arterioscler Thromb Vasc Biol 2023;43:547–561. 10.1161/ATVBAHA.122.318110 PMC10026973 36794585

[74] Goetz JG, Steed E, Ferreira RR, Roth S, Ramspacher C, Boselli F, Charvin G, Liebling M, Wyart C, Schwab Y, Vermot J. Endothelial cilia mediate low flow sensing during zebrafish vascular development. Cell Rep 2014;6:799–808. 10.1016/j.celrep.2014.01.032 24561257

[75] Parmar KM, Larman HB, Dai G, Zhang Y, Wang ET, Moorthy SN, Kratz JR, Lin Z, Jain MK, Gimbrone MA Jr, Garcia-Cardena G. Integration of flow-dependent endothelial phenotypes by Kruppel-like factor 2. J Clin Invest 2006;116:49–58. 10.1172/JCI24787 PMC1307560 16341264

[76] Packham IM, Gray C, Heath PR, Hellewell PG, Ingham PW, Crossman DC, Milo M, Chico TJ. Microarray profiling reveals CXCR4a is downregulated by blood flow in vivo and mediates collateral formation in zebrafish embryos. Physiol Genomics 2009;38:319–327. 10.1152/physiolgenomics.00049.2009 19509081

[77] Kim K, Drummond I, Ibraghimov-Beskrovnaya O, Klinger K, Arnaout MA. Polycystin 1 is required for the structural integrity of blood vessels. Proc Natl Acad Sci U S A 2000;97:1731–1736. 10.1073/pnas.040550097 PMC26504 10677526

[78] Wu G, Markowitz GS, Li L, D'Agati VD, Factor SM, Geng L, Tibara S, Tuchman J, Cai Y, Park JH, van Adelsberg J, Hou H Jr, Kucherlapati R, Edelmann W, Somlo S. Cardiac defects and renal failure in mice with targeted mutations in Pkd2. Nat Genet 2000;24:75–78. 10.1038/71724 10615132

[79] Tsiokas L, Kim E, Arnould T, Sukhatme VP, Walz G. Homo- and heterodimeric interactions between the gene products of PKD1 and PKD2. Proc Natl Acad Sci U S A 1997;94:6965–6970. 10.1073/pnas.94.13.6965 PMC21268 9192675

[80] Sharif-Naeini R, Folgering JH, Bichet D, Duprat F, Lauritzen I, Arhatte M, Jodar M, Dedman A, Chatelain FC, Schulte U, Retailleau K, Loufrani L, Patel A, Sachs F, Delmas P, Peters DJ, Honore E. Polycystin-1 and -2 dosage regulates pressure sensing. Cell 2009;139:587–596. 10.1016/j.cell.2009.08.045 19879844

[81] Kurundkar AR, Kurundkar D, Rangarajan S, Locy ML, Zhou Y, Liu RM, Zmijewski J, Thannickal VJ. The matricellular protein CCN1 enhances TGF-beta1/SMAD3-dependent profibrotic signaling in fibroblasts and contributes to fibrogenic responses to lung injury. FASEB J 2016;30:2135–2150. 10.1096/fj.201500173 PMC4871800 26884454

[82] Zhang C, Wei W, Tu S, Liang B, Li C, Li Y, Luo W, Wu Y, Dai X, Wang Y, Zheng L, Hao L, Zhang C, Luo Z, Chen YG, Yan X. Upregulation of CYR61 by TGF-beta and YAP signaling exerts a counter-suppression of hepatocellular carcinoma. J Biol Chem 2024;300:107208. 10.1016/j.jbc.2024.107208 PMC11021963 38521502

[83] Murphy-Ullrich JE, Suto MJ. Thrombospondin-1 regulation of latent TGF-beta activation: a therapeutic target for fibrotic disease. Matrix Biol 2018;68-69:28–43. 10.1016/j.matbio.2017.12.009 PMC6015530 29288716

[84] Yamashiro Y, Thang BQ, Ramirez K, Shin SJ, Kohata T, Ohata S, Nguyen TAV, Ohtsuki S, Nagayama K, Yanagisawa H. Matrix mechanotransduction mediated by thrombospondin-1/integrin/YAP in the vascular remodeling. Proc Natl Acad Sci U S A 2020;117:9896–9905. 10.1073/pnas.1919702117 PMC7211957 32321834

[85] Souilhol C, Harmsen MC, Evans PC, Krenning G. Endothelial-mesenchymal transition in atherosclerosis. Cardiovasc Res 2018;114:565–577. 10.1093/cvr/cvx253 29309526

[86] Napoli C, de Nigris F, Williams-Ignarro S, Pignalosa O, Sica V, Ignarro LJ. Nitric oxide and atherosclerosis: an update. Nitric Oxide 2006;15:265–279. 10.1016/j.niox.2006.03.011 16684613

[87] Dimmeler S, Zeiher AM. Nitric oxide-an endothelial cell survival factor. Cell Death Differ 1999;6:964–968. 10.1038/sj.cdd.4400581 10556973

[88] Lorthioir A, Joannides R, Remy-Jouet I, Freguin-Bouilland C, Iacob M, Roche C, Monteil C, Lucas D, Renet S, Audrezet MP, Godin M, Richard V, Thuillez C, Guerrot D, Bellien J. Polycystin deficiency induces dopamine-reversible alterations in flow-mediated dilatation and vascular nitric oxide release in humans. Kidney Int 2015;87:465–472. 10.1038/ki.2014.241 25029430

[89] Nauli SM, Kawanabe Y, Kaminski JJ, Pearce WJ, Ingber DE, Zhou J. Endothelial cilia are fluid shear sensors that regulate calcium signaling and nitric oxide production through polycystin-1. Circulation 2008;117:1161–1171. 10.1161/CIRCULATIONAHA.107.710111 PMC3071982 18285569

[90] Ohata S, Herranz-Perez V, Nakatani J, Boletta A, Garcia-Verdugo JM, Alvarez-Buylla A. Mechanosensory genes Pkd1 and Pkd2 contribute to the planar polarization of brain ventricular epithelium. J Neurosci 2015;35:11153–11168. 10.1523/JNEUROSCI.0686-15.2015 PMC4524982 26245976

[91] Dinsmore C, Reiter JF. Endothelial primary cilia inhibit atherosclerosis. EMBO Rep 2016;17:156–166. 10.15252/embr.201541019 PMC5290813 26769565

