# Supplementary material for: Polycystin-1 suppresses apoptotic signalling in endothelial cells and protects from atherosclerosis
Source: Cardiovasc Res. 2026 Aug 10;122(14):1922–35. doi: 10.1093/cvr/cvag171 (PMC13653497; doi:10.1093/cvr/cvag171)
Supplement: Supplementary file 1 [file cvag171_supplementary_data.zip › Tardajos et al_Supplement.pdf]

Supplementary Material

Supp. Figures 1-18

Supp. Tables 1-9

Supp. References

Supp. Figure 1

A

HUMAN GENE	MECHANORECEPTOR TYPE	FISH ORTHOLOGUE (Ensembl GENE ID)	EC EXPRESSION
BDKRB2	RECEPTOR	ENSDARG00000043664	NO
CAV1	SCAFFOLD/CAVEOLAE	ENSDARG00000103747	YES
CDH5	ADHESION	ENSDARG00000075549	YES
GPC1	PROTEOGLYCAN/GLYCOCALYX	ENSDARG00000019341 (*gpc1a*)<br>ENSDARG00000090585 (*gpc1b*)	NO<br>NO
ITGA5	INTEGRIN	ENSDARG00000006353	YES
ITGB1	INTEGRIN	ENSDARG00000071863 (*itgb1a*)<br>ENSDARG00000104484 (*itgb1b*)<br>ENSDARG00000053232 (*itg1b.1*)<br>ENSDARG00000022689 (*itg1b.2*)	YES<br>YES<br>YES<br>NO
PECAM1	ADHESION	ENSDARG00000060263	YES
PIEZO1	ION CHANNEL	ENSDARG00000076870	YES
PKD1	GLYCOPROTEIN/CILIA	ENSDARG00000030417 (*pkd1a*)<br>ENSDARG00000033029 (*pkd1b*)	YES<br>NO
PKD2	ION CHANNEL/CILIA	ENSDARG00000014098	YES
SDC4	PROTEOGLYCAN	ENSDARG00000059906	YES
TRPV4	ION CHANNEL	ENSDARG00000061941	NO

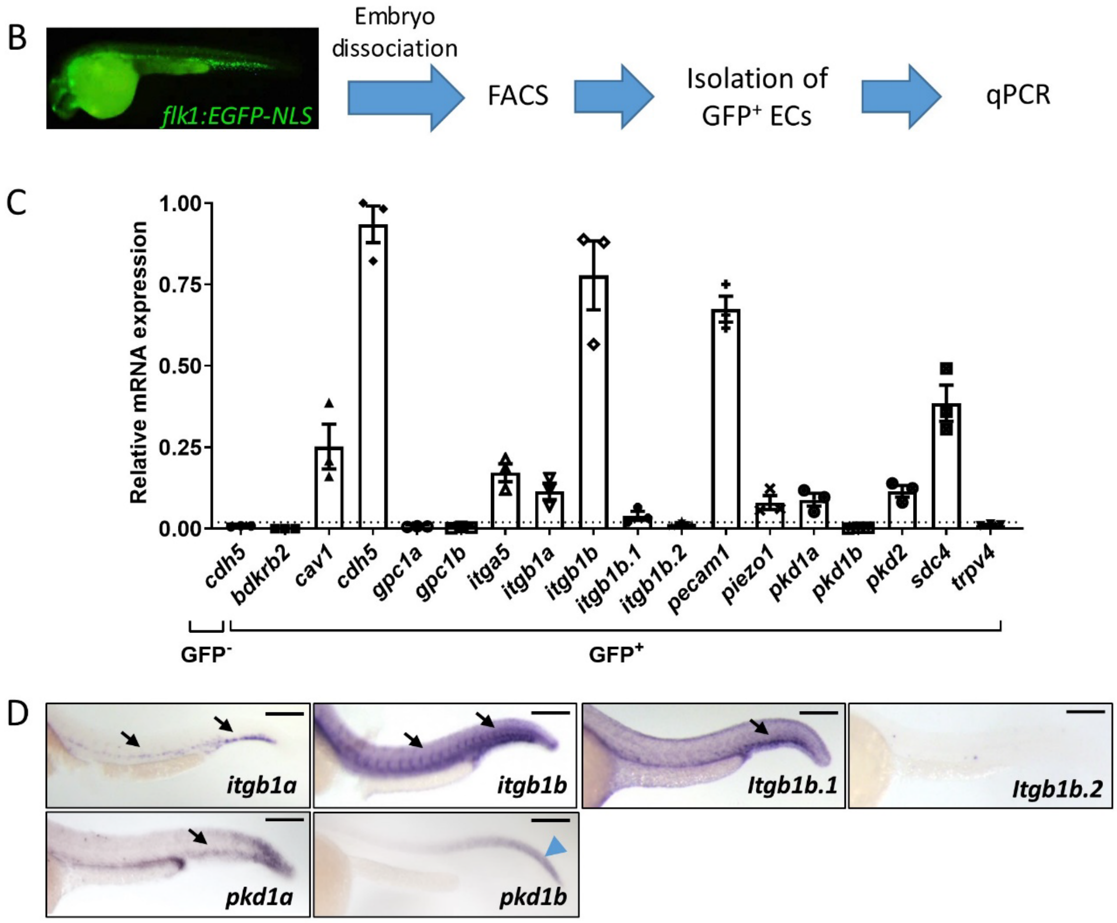

Supp. Fig. 1. Selection of candidate endothelial mechanoreceptors for functional screening in zebrafish embryos. A Table showing candidate known and putative endothelial cell (EC) mechanoreceptors selected for functional screening in zebrafish embryos. **B** Schematic diagram

showing workflow for fluorescence activated cell sorting (FACS) of Tg(*flk1a:EGFP-NLS*) embryos to isolate GFP$^+$ ECs for gene expression analysis. Adapted from Serbanovic-Canic et al.[31] **C** Relative gene expression analysis of GFP$^+$ ECs isolated from 30 hours post fertilization (hpf) old zebrafish embryos using qPCR and *bact2* as a housekeeping gene. Genes analysed are indicated on the *x*-axis. GFP$^-$ cells were used as a negative control. N=3 with 300-400 embryos per experiment. **D** Whole-mount *in situ* hybridisation analysis of mRNA expression levels in Tg(*flk1a:EGFP-NLS*) embryos at 30 hpf. Black arrows indicate EC expression of *itgb1a*, *itgb1b*, *itgb1b.1* and *pkd1a*, while *itgb1b.2* and *pkd1b* showed no EC expression. *Itgb1a* showed endothelial-specific expression, whereas *itgb1b* and *itgb1b.1* were more ubiquitously expressed (purple staining). *Pkd1b* was expressed in the nervous system as indicated by blue arrowhead. Scale bar: D, 500 μm.

Supp. Figure 2

A

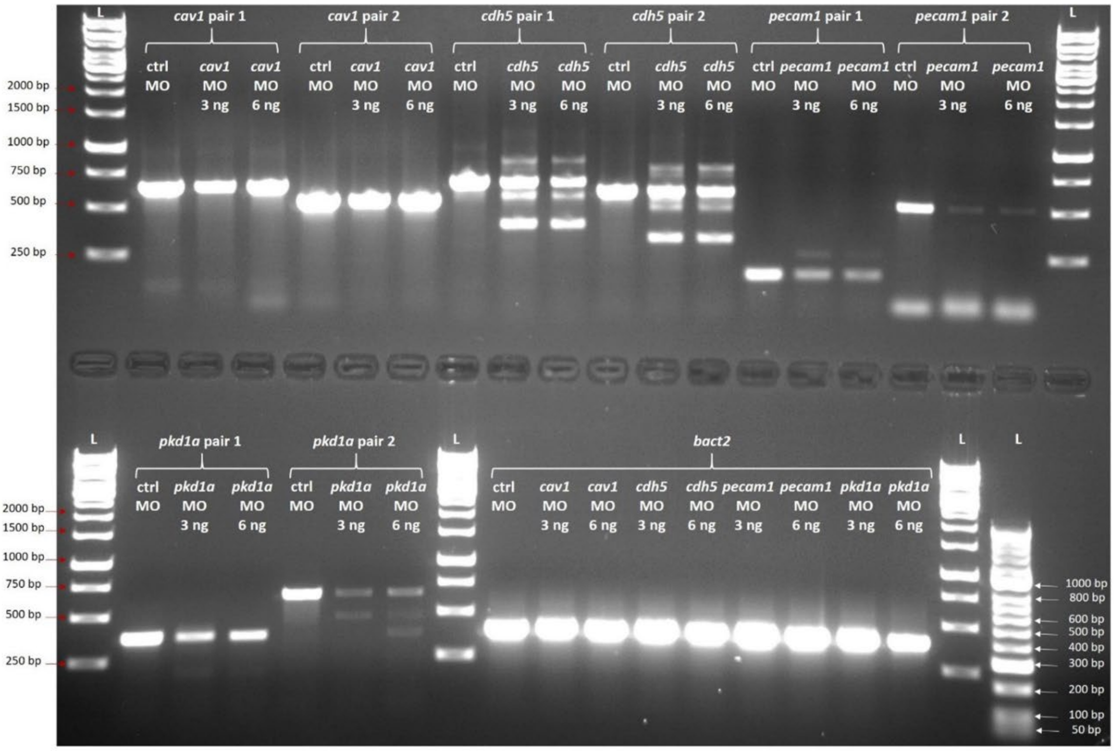

B

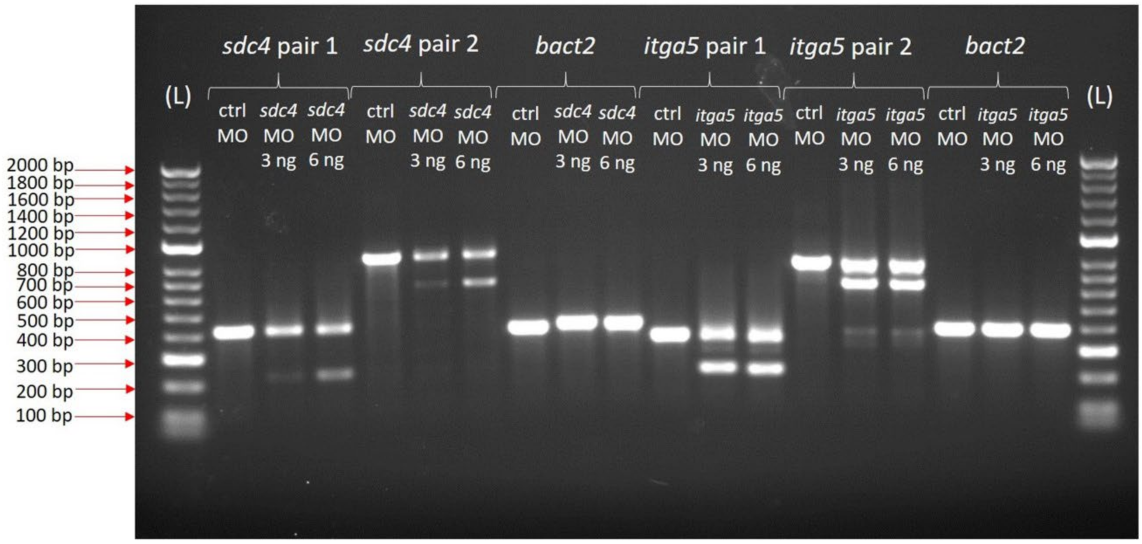

Supp. Fig. 2. Validation of morpholino oligonucleotide (MO) efficiency by RT-PCR. A-B Zebrafish Tg(*flk1a:EGFP-NLS*) were injected with 3 ng or 6 ng of gene-targeting MOs or 6 ng of negative control non-targeting MO, and total RNA isolated at 30 hours post fertilisation (hpf). The ability of gene-specific splice-blocking MOs to modify splicing of targeted transcripts was assessed by analysis of RT-PCR products after agarose gel electrophoresis, using primers flanking the targeted regions. Targeted genes are indicated in the gel images. Splicing modifications were observed as a band-shift or as a reduction

of the wildtype band compared to the control sample (ctrl MO). RT-PCR analysis of beta-actin (*bact2*) was used as an internal control. The DNA fragment sizes are indicated in base pairs (bp) next to the DNA ladder (L).

Supp. Figure 3

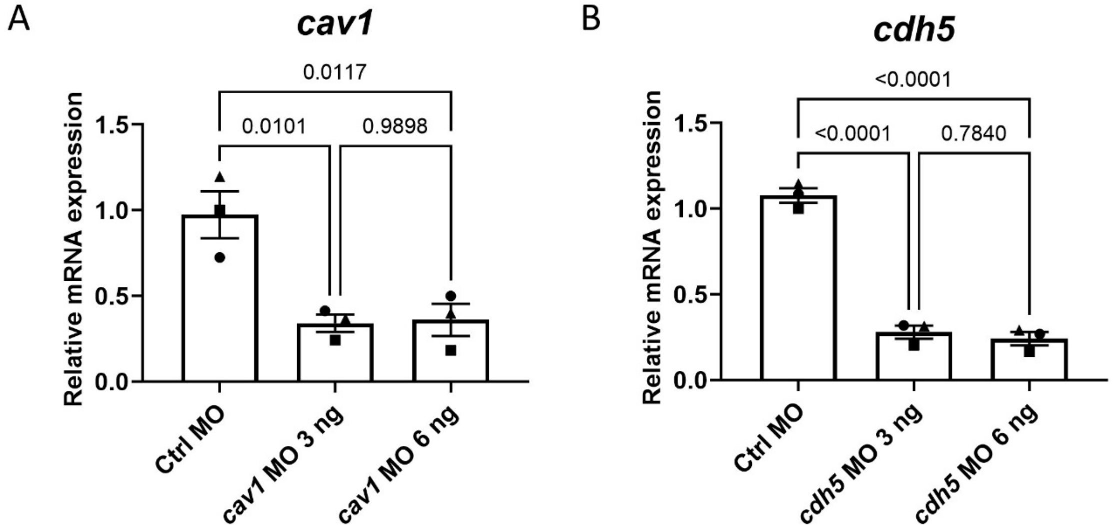

Supp. Fig. 3. Validation of morpholino oligonucleotide (MO) efficiency by qPCR. A-B Zebrafish Tg(*flk1a:EGFP-NLS*) were injected with 3 ng or 6 ng of gene-targeting MOs or 6 ng of negative control non-targeting MO, and total RNA was isolated at 30 hours post fertilisation (hpf). MOs that showed residual expression of the targeted wildtype transcript by RT-PCR (*cav1*, *cdh5* in Supp. Fig. 2) were further tested by qPCR. Based on sequencing results of the additional bands produced by MO injection, the qPCR primers were designed to detect the wildtype transcript only. N=3 with 15-20 embryos per experiment. Differences between groups were analysed using one-way ANOVA with Tukey's post-hoc test (A-B), with p-values shown in the graphs.

Supp. Figure 4

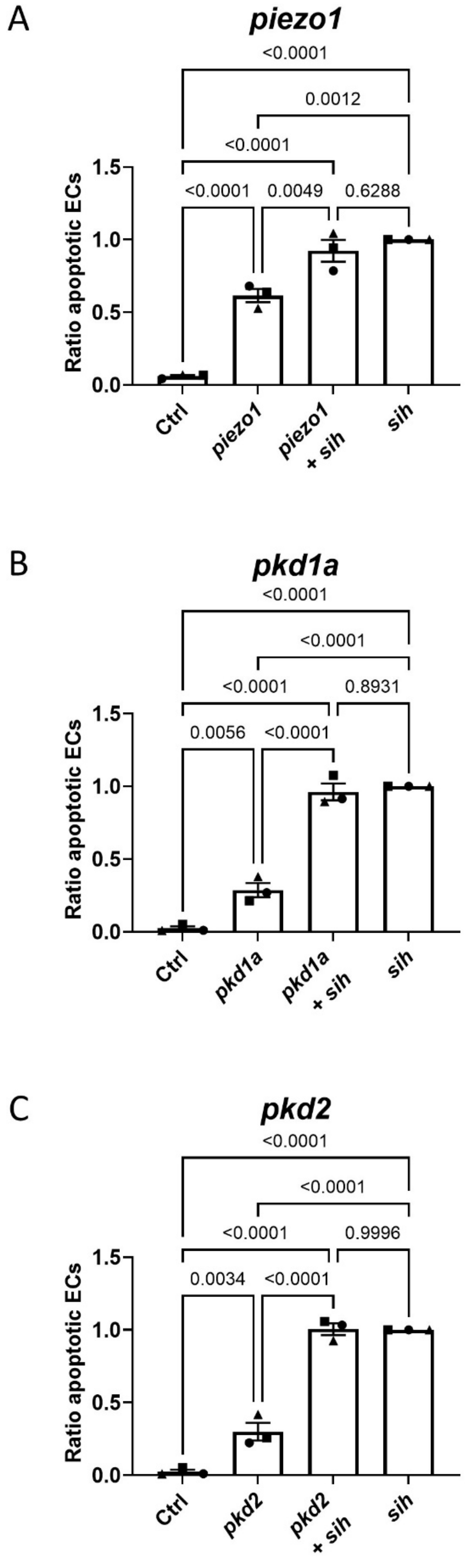

Supp. Fig. 4. *Piezo1, pkd1a* and *pkd2* protect from endothelial cell (EC) apoptosis in a flow-dependent manner. A-C Zebrafish transgenic Tg(*flk1:EGFP-NLS*) embryos were injected with morpholino oligonucleotide (MO) targeting *piezo1* (A), *pkd1a* (B) or *pkd2* (C) with or without the *silent*

heart (*sih*) MO to prevent blood flow. Non-targeting control MO was used as a negative control. EC apoptosis was studied by whole-mount active caspase-3 staining of 30 hpf embryos. The proportion of apoptotic ECs (number of apoptotic ECs divided by the total number of ECs) normalised to *sih* MO-injected embryos was calculated and is presented in the graphs. Each data point represents an independent experiment with n≥5 embryos. Differences between groups were analysed using a one-way ANOVA with Tukey's post-hoc test, and p-values are shown in the graphs.

Supp. Figure 5

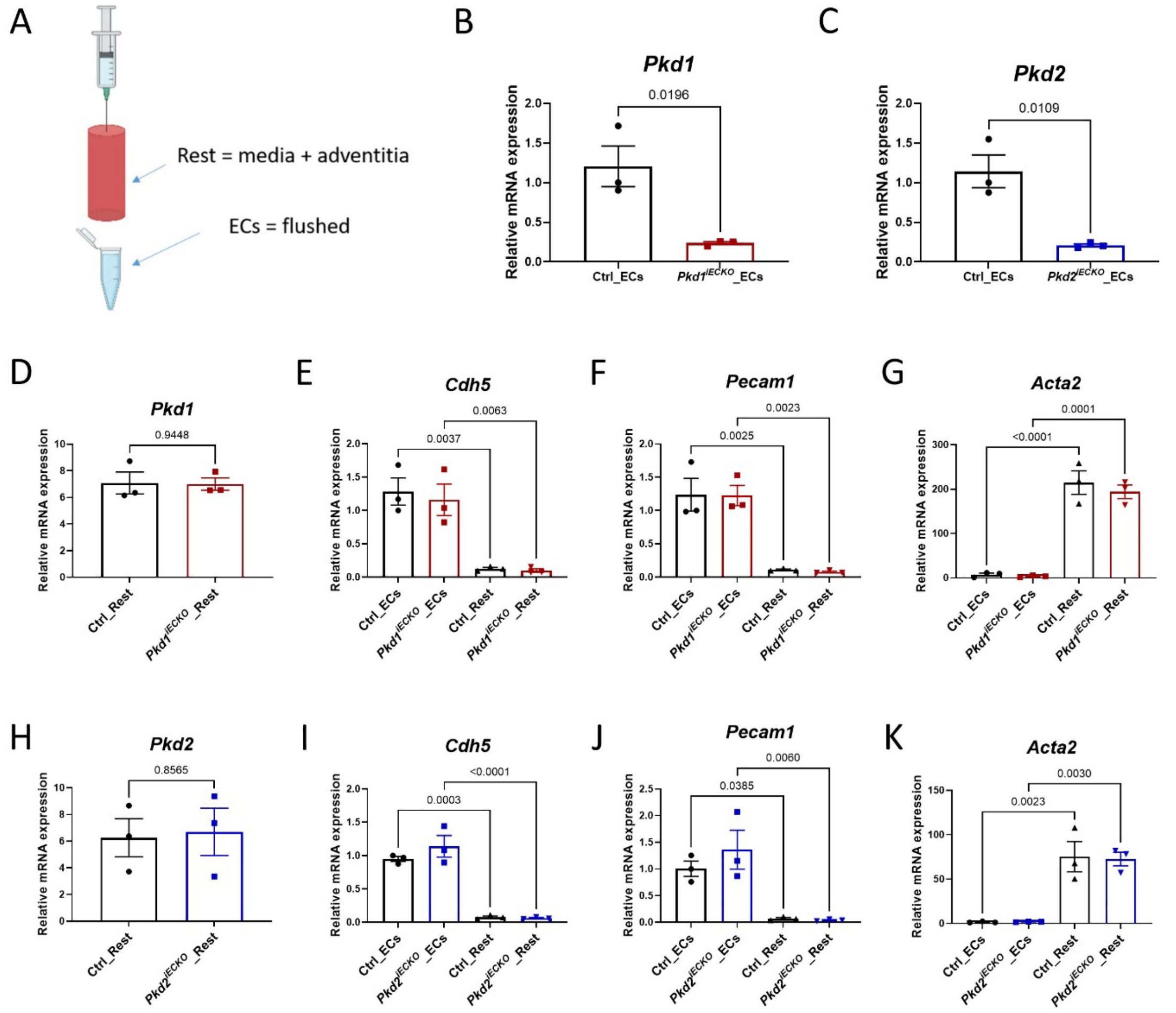

Supp. Fig. 5. Validation of endothelial cell (EC)-specific, inducible *Pkd1* and *Pkd2* deletion in *Pkd1*^{iECKO} and *Pkd2*^{iECKO} mice, respectively. A Schematic diagram showing flushing of mouse aortas with QIAzol reagent to obtain flushed samples enriched in intimal ECs, while the rest of the aorta, containing media and adventitia, was used as a negative control. **B-K** Mice (*Pkd1*$^{fl/fl}$ *Cdh5*$^{Cre\text{-}ERT2/+}$ or *Pkd1*$^{fl/fl}$ *Cdh5*$^{+/+}$ littermate controls shown in B, D-G and *Pkd2*$^{fl/fl}$ *Cdh5*$^{Cre\text{-}ERT2/+}$ or *Pkd2*$^{fl/fl}$ *Cdh5*$^{+/+}$ littermate controls shown in C, H-K) were injected with tamoxifen for 5 consecutive days to activate Cre and generate *Pkd1*^{iECKO} and *Pkd2*^{iECKO} mice, or control mice, respectively. Intimal aortic EC samples were collected by flushing after two weeks. Gene expression was analysed by qPCR of total RNA isolated from flushed, EC-enriched samples and the aorta after the flushing. **B** Expression of *Pkd1* was significantly decreased in EC samples from *Pkd1*^{iECKO} mice compared to control mice (n=3 mice per group). **C** Expression of *Pkd2* was significantly decreased in EC samples from *Pkd2*^{iECKO} mice compared to control mice (n=3 mice per group). **D-G** Expression of *Pkd1*, endothelial markers *Cdh5* and *Pecam1*, and vascular smooth muscle cell (VSMC) marker *Acta2* was measured in EC samples and the remaining aortic samples of *Pkd1*^{iECKO} and control mice after intimal EC flushing (Rest) by qPCR (n=3 mice per group). **D** *Pkd1* expression in VSMCs was not affected by EC-specific Cre activation. **E-**

F EC-specific markers *Cdh5* and *Pecam1* were significantly upregulated in EC-enriched intimal samples (ECs) compared to the rest of the aorta (Rest). **G** VSMC marker *Acta2* was significantly upregulated in the rest of the aorta (Rest) compared to EC-enriched intimal samples (ECs). **H-K** Expression of *Pkd2*, endothelial markers *Cdh5* and *Pecam1*, and vascular smooth muscle cell (VSMC) marker *Acta2* was measured in EC samples and the remaining aortic samples of $Pkd2^{iECKO}$ and control mice after intimal EC flushing (Rest) by qPCR (n=3 mice per group). **H** *Pkd2* expression in VSMCs was not affected by EC-specific Cre activation. **I-J** EC-specific markers *Cdh5* and *Pecam1* were significantly upregulated in EC-enriched intimal samples (ECs) compared to the rest of the aorta (Rest). **K** VSMC marker *Acta2* was significantly upregulated in the rest of the aorta (Rest) compared to EC-enriched intimal samples (ECs). Differences between groups were analysed using upaired t-test (B, C) or a two-way ANOVA with Tukey's post-hoc test, and p-values are shown in the graphs.

Supp. Figure 6

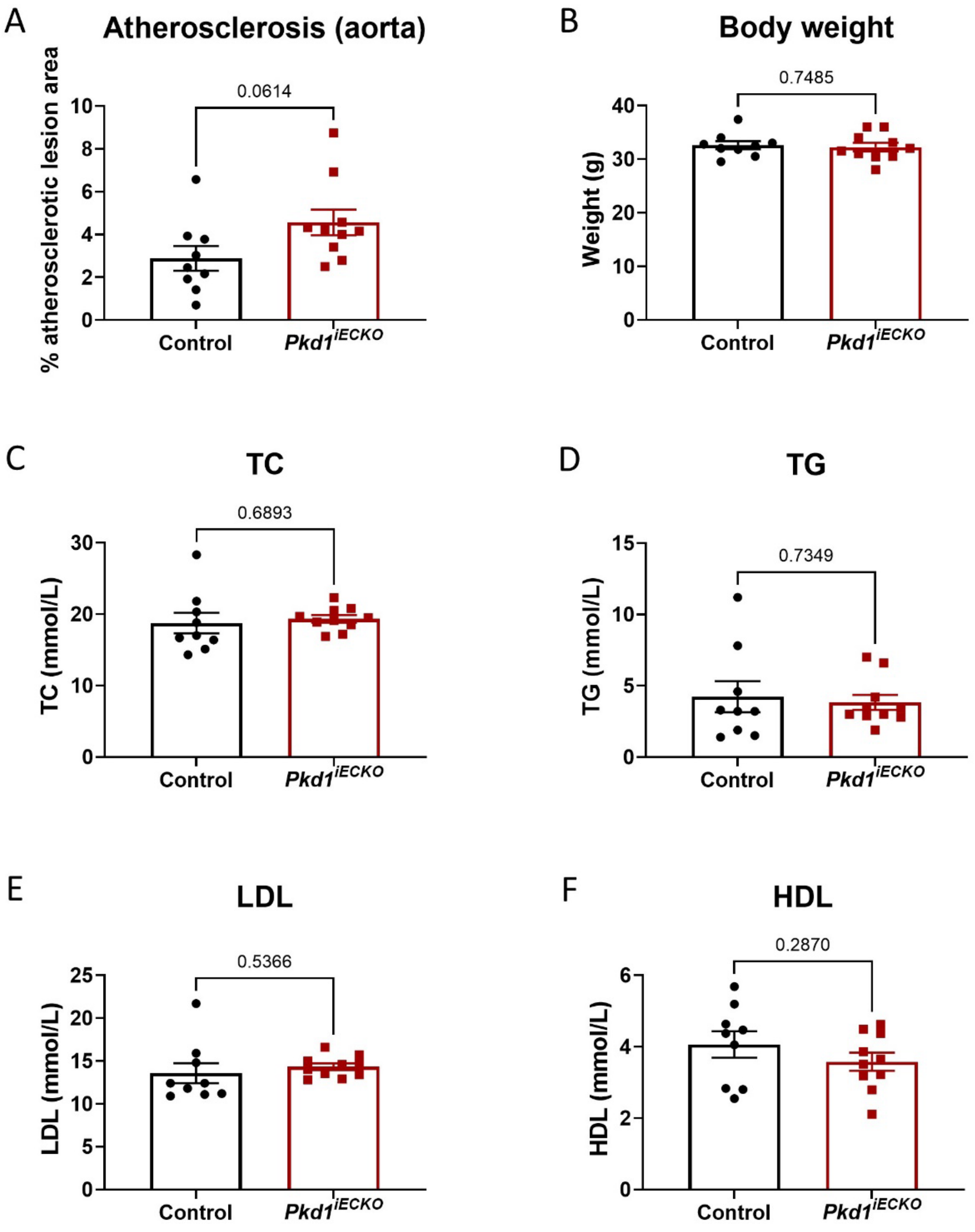

Supp. Fig. 6. Plaque analysis, weight and lipid profile of *Pkd1^{iECKO}* and control mice. *Pkd1$^{fl/f}$ Cdh5$^{CreERT2/+}$* (*Pkd1^{iECKO}*) mice aged 6 weeks and littermates lacking Cre (*Pkd1$^{fl/fl}$ Cdh5$^{+/+}$*; controls) received five intraperitoneal injections of tamoxifen and one intravenous injection of AAV-PCSK9 two weeks after first tamoxifen injection. Following 6 weeks of feeding with high-fat diet (HFD), the mice were culled and plasma samples and aortas collected for analysis. **A** Quantification of plaque burden in the aorta was determined by calculating the percentage of aortic surface area covered by plaque for *Pkd1^{iECKO}* mice (n = 10) and littermate controls (n = 9). **B-F** There was no significant difference in body weight (B), total cholesterol – TC (C), triglycerides – TG (D), low density lipoprotein – LDL (E) and high density lipoprotein – HDL (F) between *Pkd1^{iECKO}* and control mice. Differences between groups were analysed using upaired t-test (A-F), and p-values are shown in the graphs.

Supp. Figure 7

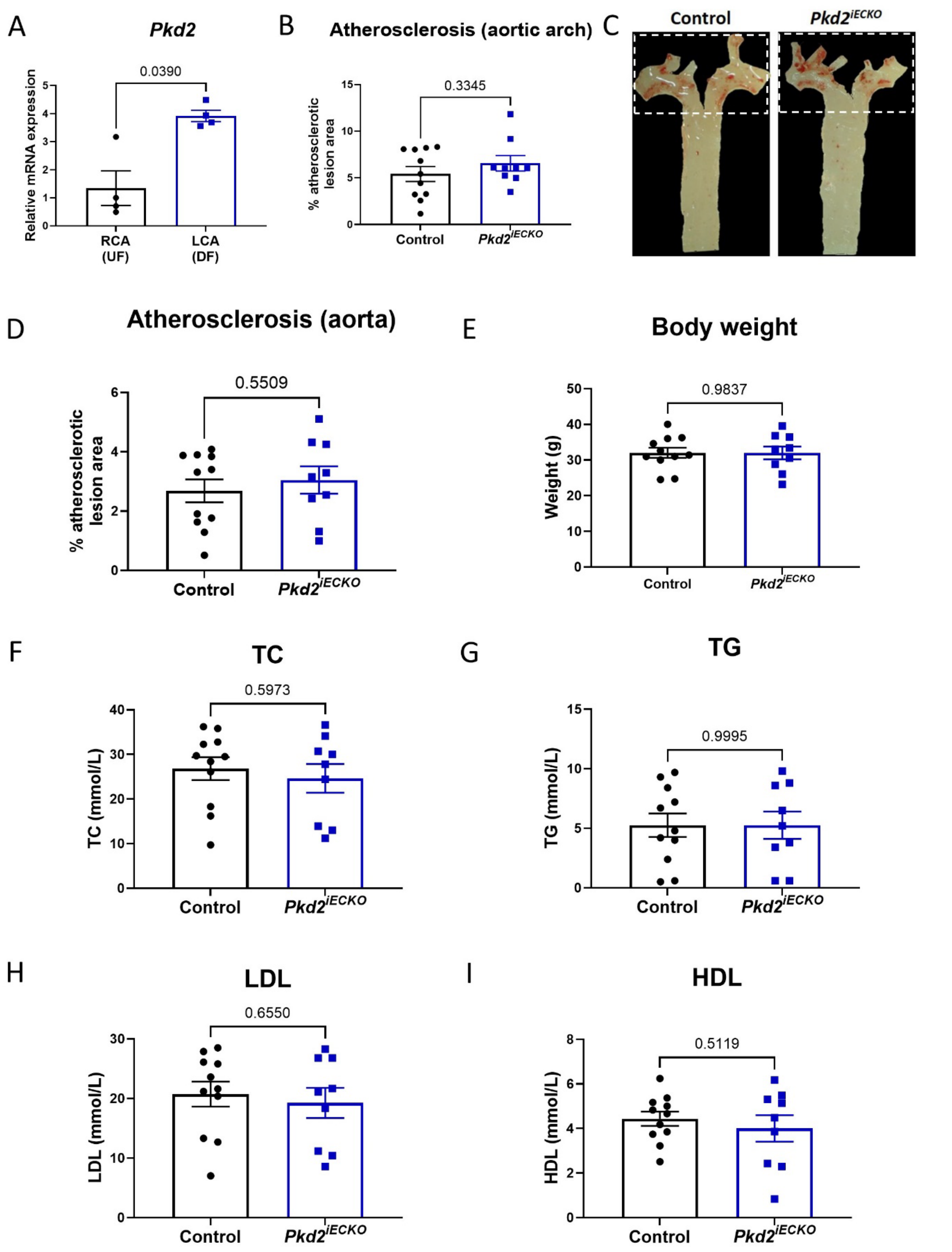

Supp. Fig. 7. Expression analysis of *Pkd2* mRNA expression in the partially ligated carotid arteries and plaque analysis, weight and lipid profile of *Pkd2*^{iECKO} and control mice. A *Pkd2* mRNA

expression is induced by disturbed flow (DF) conditions. C57BL/6 mice at 8 weeks of age (n=4) were subjected to partial ligation of the left carotid artery (LCA), whereas the right carotid artery (RCA) was sham-operated as a baseline control. Intimal RNA from LCA exposed to disturbed flow (DF) and RCA exposed to undisturbed flow (UF) was collected 2 days after surgery. Relative *Pkd2* mRNA expression was measured by qPCR. **B-I** $Pkd2^{fl/f}$ $Cdh5^{CreERT2/+}$ ($Pkd2^{iECKO}$) mice aged 6 weeks and littermates lacking Cre ($Pkd2^{fl/fl}$ $Cdh5^{+/+}$; controls) received five intraperitoneal injections of tamoxifen and one intravenous injection of AAV-PCSK9 two weeks after first tamoxifen injection. Following 6 weeks of feeding with high-fat diet (HFD), the mice were culled and plasma samples and aortas collected for analysis. **B** Quantification of plaque burden in the aortic arch was determined by calculating the percentage of aortic surface area covered by plaque for $Pkd2^{iECKO}$ mice (n = 9) and littermate controls (n = 11). **C** Representative images of aortas isolated from $Pkd2^{iECKO}$ and control mice stained with Oil Red O. Aortic arch is indicated by white dashed line. **D** Quantification of plaque burden in the aorta was determined by calculating the percentage of aortic surface area covered by plaque for $Pkd2^{iECKO}$ mice (n = 9) and littermate controls (n = 11). **E-I** There was no significant difference in body weight (E), total cholesterol – TC (F), triglycerides – TG (G), low density lipoprotein – LDL (H) and high density lipoprotein – HDL (I) between $Pkd2^{iECKO}$ and control mice. Differences between groups were analysed using unpaired t-test (A-B, D-I), and p-values are shown in the graphs.

Supp. Figure 8

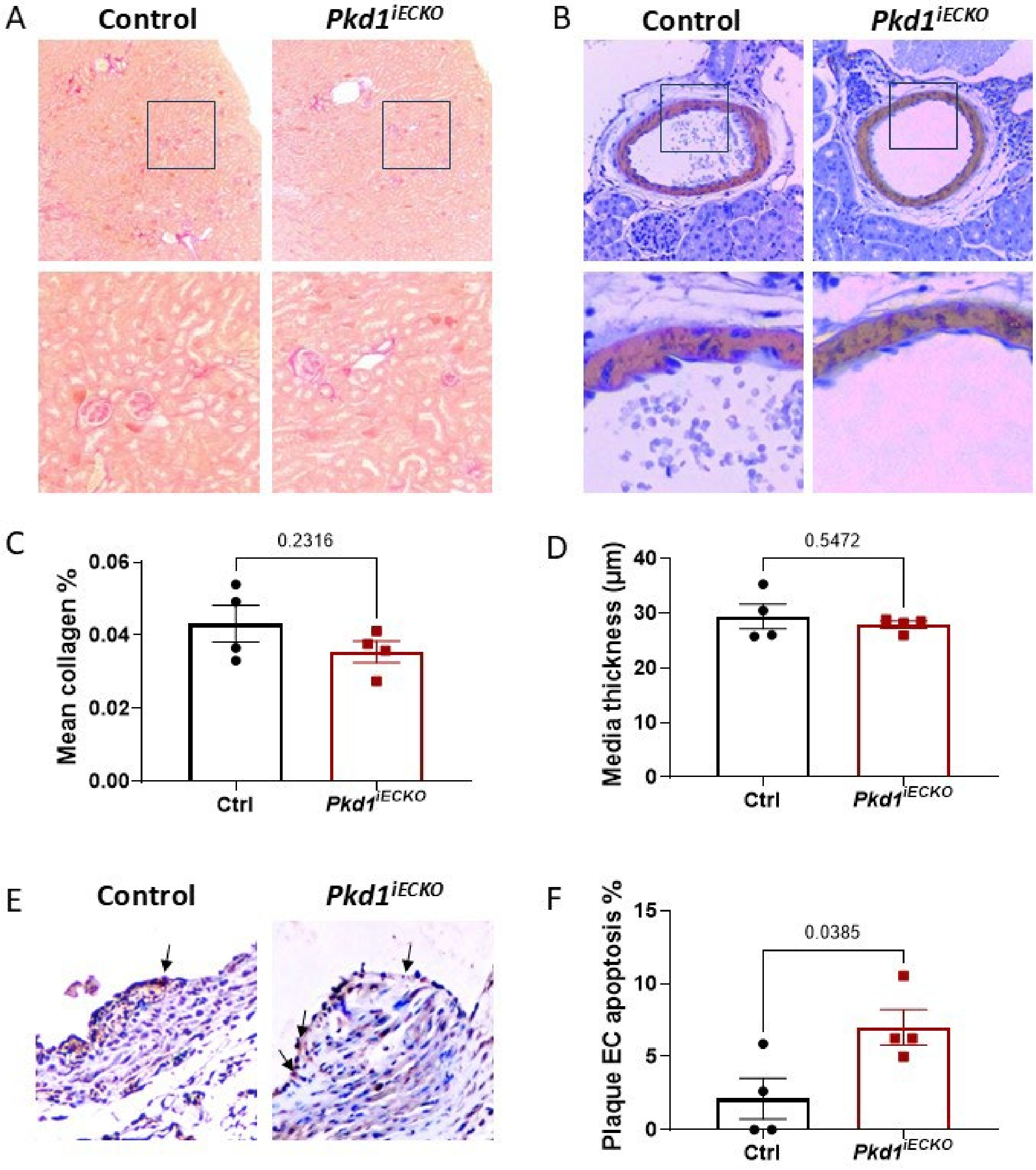

Supp. Fig. 8. Immunohistochemical analysis of kidneys and aortic roots. **A-D** Kidneys from *Pkd1*^{iECKO} and control mice were isolated 6 weeks following high fat diet feeding and stained with Picrosirius Red to assess renal fibrosis (C) or an ACTA2 antibody to assess media thickness of medium-sizes arteries (D). **E-F** Aortic roots from *Pkd1*^{iECKO} and control mice were isolated 6 weeks following high fat diet feeding and stained using colorimetric TUNEL kit to assess apoptosis of endothelial cells overlying atherosclerotic plaques. Differences between groups were analysed using upaired t-test (C, D, F), and p-values are shown in the graphs.

Supp. Figure 9

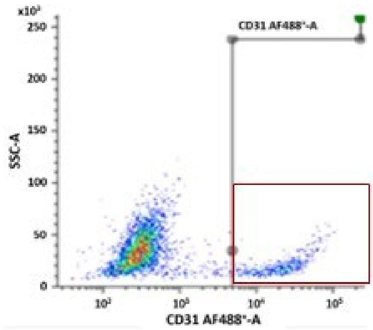

Supp. Fig. 9. Single cell RNA sequencing (scRNAseq) analysis of mouse aortic endothelial cells (ECs) isolated from *$Pkd1^{iECKO}$* and control mice. *$Pkd1^{fl/fl}$ $Cdh5^{Cre\text{-}ERT2/+}$* or *$Pkd1^{fl/fl}$ $Cdh5^{+/+}$* littermate controls were injected with tamoxifen for 5 consecutive days to activate Cre and generate *$Pkd1^{iECKO}$* and control mice, respectively. Two weeks later, aortas from *$Pkd1^{iECKO}$* and control mice (n=4 experiments) were processed to generate single cells and then analysed by fluorescence-activated cell sorting (FACS) of $CD31^{+}$ $CD45^{-}$ cells coupled to scRNAseq. Representative flow cytometry profile of CD31 staining after gating out $CD45^{+}$ cells. $CD31^{+}$ cells were sorted into 384-well plate for scRNAseq.

Supp. Figure 10

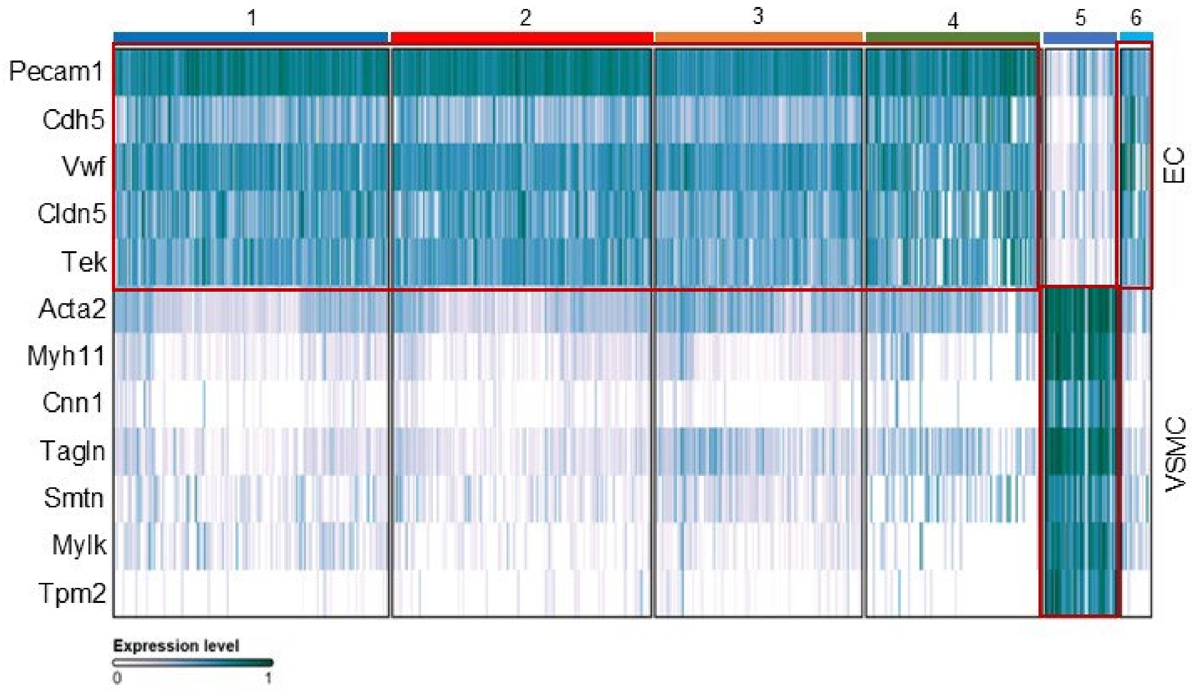

Supp. Fig. 10. scRNA-seq identification of EC subsets. Aortas from *Pkd1*^{iECKO} and control mice were analysed by FACS of CD31^{+} CD45^{-} cells coupled to scRNA-seq. Heatmap showing the expression of canonical endothelial (EC) and vascular smooth muscle cell (VSMC) markers. Five distinct clusters (1-4 and 6) defined as ECs exhibit common expression EC markers. Cluster 5 expresses several canonical VSMC markers including Myh11, Acta2, and Cnn1 and were therefore defined as VSMCs.

Supp. Figure 11

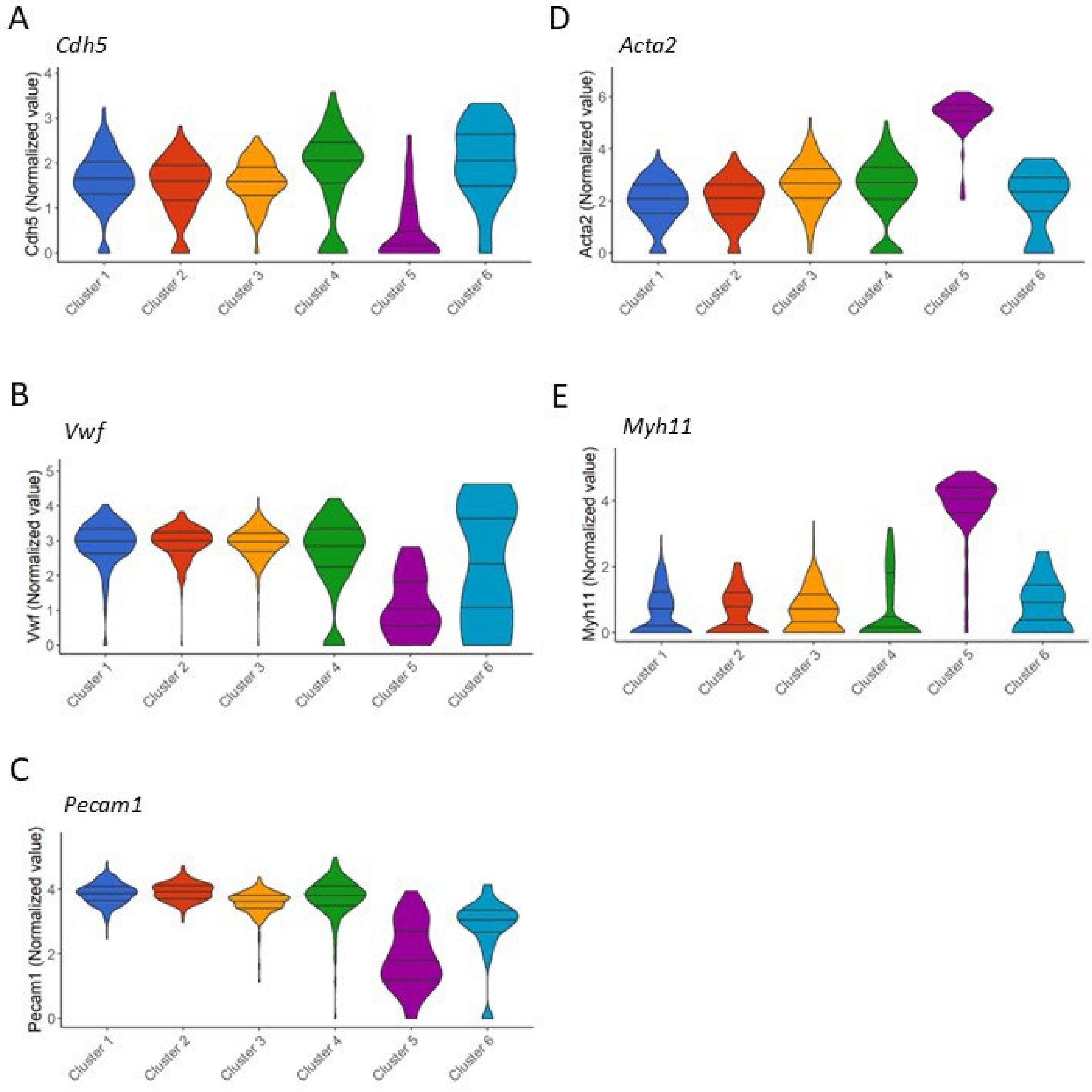

Supp. Fig. 11. Relative expression analysis of endothelial cell (EC) and vascular smooth muscle cell (VSMC) markers in clusters 1-6 identified by single cell RNA sequencing (scRNAseq) of mouse aortic ECs isolated from *Pkd1^{iECKO}* and control mice. *Pkd1$^{fl/fl}$ Cdh5$^{Cre\text{-}ERT2/+}$* or *Pkd1$^{fl/fl}$ Cdh5$^{+/+}$* littermate controls were injected with tamoxifen for 5 consecutive days to activate Cre and generate *Pkd1^{iECKO}* and control mice, respectively. Two weeks later, aortas from *Pkd1^{iECKO}* and control mice (n=4 experiments) were processed to generate single cells and then analysed by fluorescence-activated cell sorting (FACS) of CD31^{+} CD45^{-} cells coupled to scRNAseq. **A-E** Violin plots showing relative expression of canonical EC markers *Cdh5* (A), *Vwf* (B) and *Pecam1* (C) or canonical VSMC markers *Acta2* (D) and *Myh11* (E). Five distinct clusters (1-4 and 6) were enriched for EC markers whereas cluster 5 was enriched for VSMC markers.

Supp. Figure 12

A

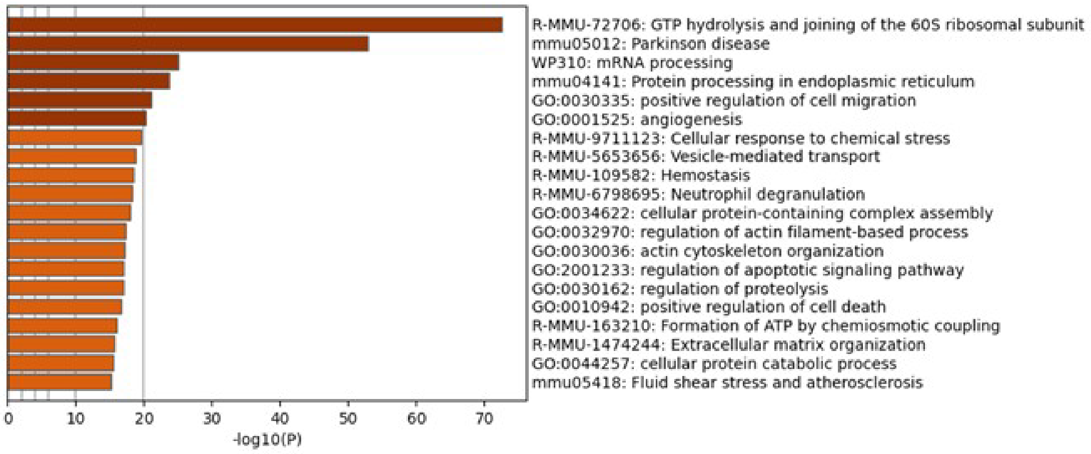

B

Gene symbol	Gene name	Fold change	P-value	EC apoptosis	Role in atherosclerosis	Inhibits NO signalling
Thbs1	Thrombospondin 1	6.88	3.91E-52	Pro-apoptotic	Pro-atherogenic	Yes
Ccn1	Cellular Communication Network Factor 1	6.03	4.08E-45	Pro-apoptotic	Pro-atherogenic	Yes
Bmp4	Bone Morphogenetic Protein 4	4.28	1.46E-61	Pro-apoptotic	Pro-atherogenic	Yes
Txndc5	Thioredoxin Domain Containing 5	2.79	1.03E-34	Anti-apoptotic	Pro-atherogenic	Yes

C

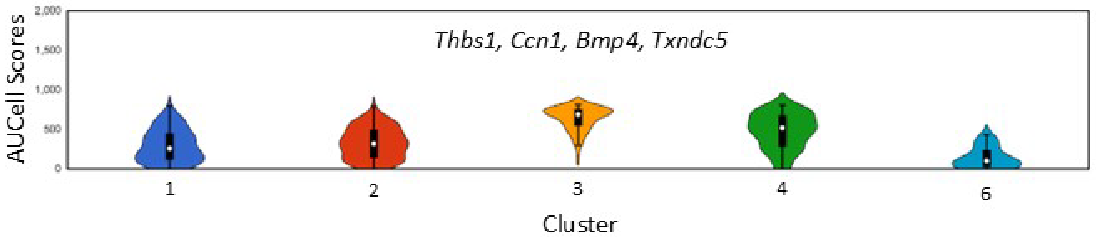

Supp. Fig. 12. Process enrichment analysis and candidate PKD1 downstream targets identified by single cell RNA sequencing (scRNAseq) of mouse aortic endothelial cells (ECs) isolated from *Pkd1*^{iECKO} and control mice. A Process enrichment analysis of genes differentially expressed in cluster 3 compared to EC clusters 1,2,4 and 6 was performed using Metascape (https://metascape.org/). **B** Table showing 4 differentially upregulated genes in *Pkd1*-regulated EC cluster 3, compared to EC clusters 1, 2, 4 and 6: *Thbs1*, *Ccn1*, *Bmp4* and *Txndc5*, which were among top twenty most upregulated genes in this cluster. They were selected for validation studies as potential downstream targets of PKD1 based on the following: 1. Known role in regulating EC apoptosis. 2. Known to promote atherosclerosis development. 3. Known role in modulation of nitric oxide signaling (references in the main text). **C** Violin plots indicating the enrichment of a gene set consisting of *Thbs1*, *Ccn1*, *Bmp4* and *Txndc5* within each cluster.

Supp. Figure 13

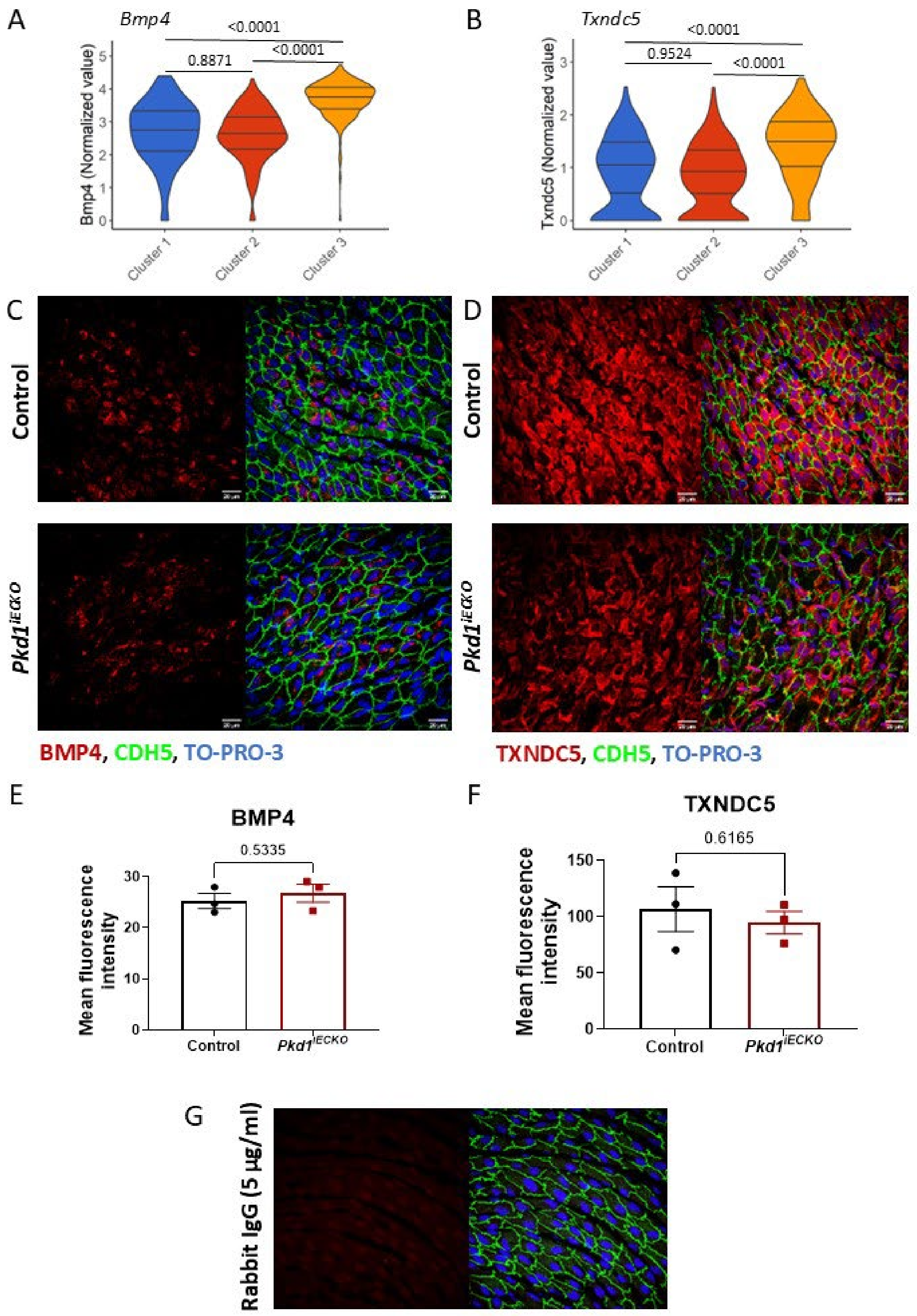

Supp. Fig. 13. Validation of candidate PKD1 downstream targets identified by single cell RNA sequencing (scRNAseq) of mouse aortic endothelial cells (ECs) isolated from *Pkd1*^{iECKO} and

control mice. A-B *Pkd1$^{fl/fl}$ Cdh5$^{Cre\text{-}ERT2/+}$* or *Pkd1$^{fl/fl}$ Cdh5$^{+/+}$* littermate controls were injected with tamoxifen for 5 consecutive days to activate Cre and generate *Pkd1^{iECKO}* and control mice, respectively. Two weeks later, aortas from *Pkd1^{iECKO}* and control mice were processed to generate single cells and then analysed by fluorescence-activated cell sorting (FACS) of CD31^{+} CD45^{-} cells coupled to scRNAseq. Violin plots indicating expression distribution of *Bmp4* (A) and *Txndc5* (B) in clusters 1, 2 and 3 identified by single cell RNA sequencing of *Pkd1*-deficient and control mouse aortic ECs. **C-D** Aortic arches were isolated from 8 week-old *Pkd1^{iECKO}* and control mice and *en face* immunostainings were performed using antibodies against BMP4 (C) and TXNDC5 (D) (red). Endothelium was co-stained with anti-CDH5 antibody (green) and nuclei were detected using TO-PRO-3 (blue). Images show inner curvature, athero-prone regions of the aortic arches. **E-F** Quantification of relative protein expression in the inner curvature of the aortic arches expressed as mean fluorescence intensity. **G** Isotype control staining. N=3 mice per group. Differences between groups were analysed using unpaired t-test with p-values shown in the graphs. Scale bars: D-E 20 µm.

Supp. Figure 14

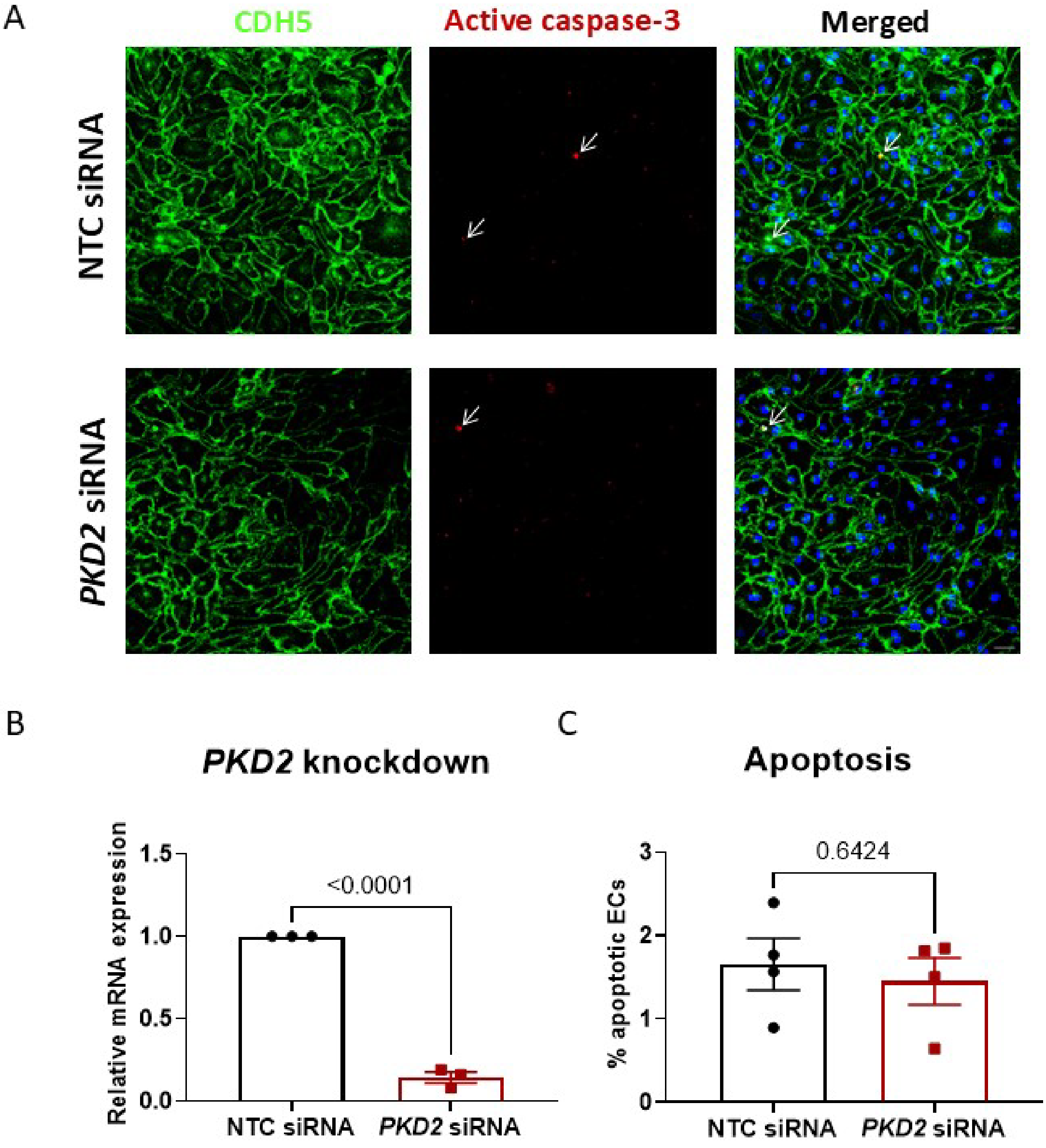

Supp. Fig. 14. Knockdown of *PKD2* in human aortic endothelial cells (HAoECs). A-C HAoECs were treated with 10 nM *PKD2* siRNA or with non-targeting control (NTC) siRNA for 72 hours. The efficiency of *PKD2* siRNA was assessed by qPCR (n=3 donors) using *HPRT* as a housekeeping gene (B). Endothelial cell (EC) apoptosis was assessed by immunostaining using anti-active caspase-3 antibody (red) and co-staining with EC marker CDH5 (green) and DAPI (blue) (A). Quantification of EC apoptosis in *PKD2* knockdown and control HAoECs (C). The graph represents percentage apoptotic ECs calculated by dividing the number of active caspase-3-positive cells by the total number of EC per field of view (n=4 donors).

Supp. Figure 15

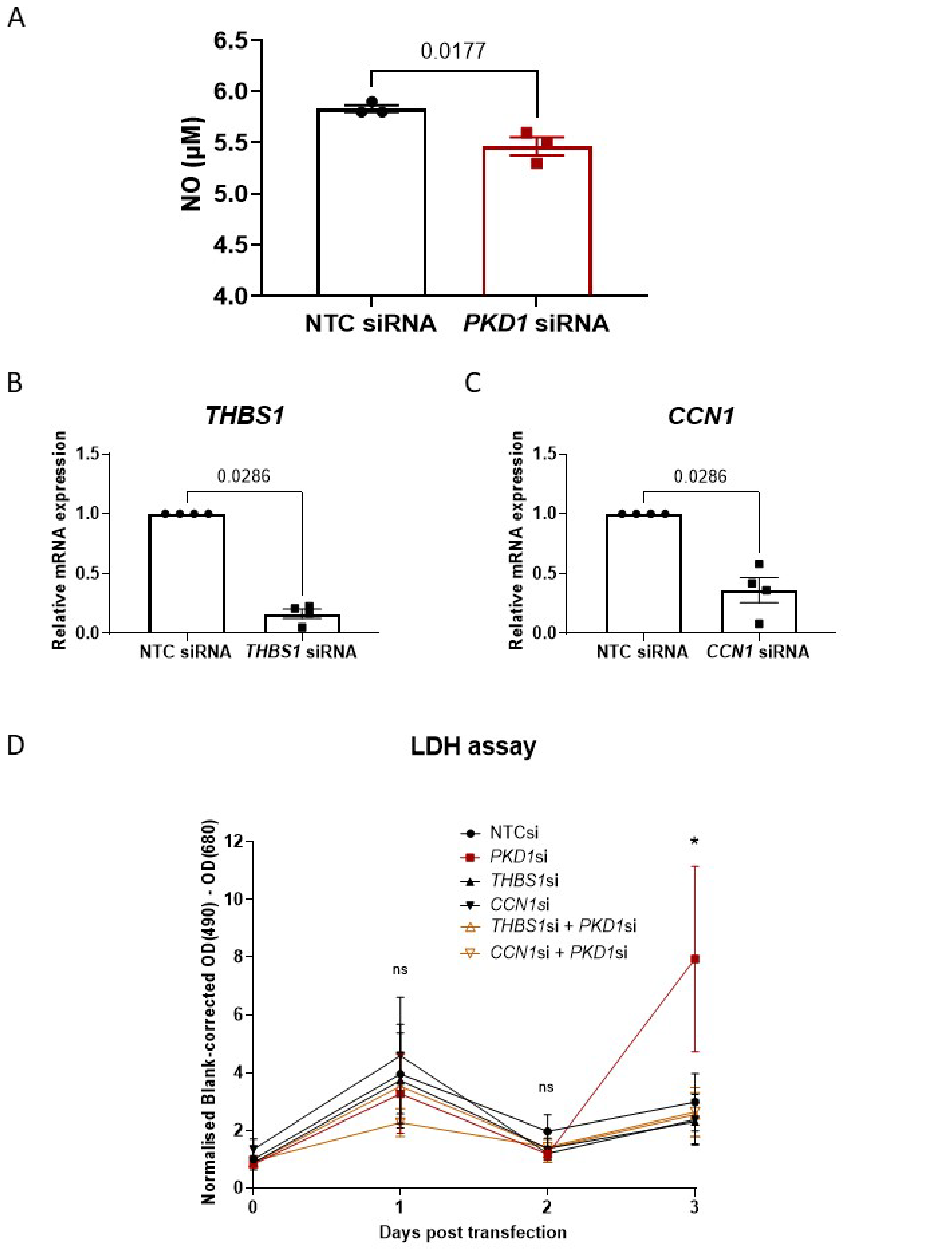

Supp. Fig. 15. Nitric oxide (NO) assay, validation of siRNA-mediated knockdown of *THBS1* and *CCN1* in human aortic endothelial cells (HAoECs) and assessment of the effect of THBS1 and CCN1 on PKD1-mediated cell death using lactate dehydrogenase (LDH) assay. A HAoECs were treated with 10 nM *PKD1* siRNA or with non-targeting control (NTC) siRNA for 72 hours. NO levels were

measured in the cell media using a commercial colorimetric kit. **B-C** HAoECs were treated with 10 nM *THBS1, CCN1* siRNA or with non-targeting control (NTC) siRNA for 72 hours. The efficiency of siRNA-mediated knockdown of *THBS1* (A) or *CCN1* (B) was assessed by qPCR (n=4 donors) using *HPRT* as a housekeeping gene. **D** HAoECs were treated with *PKD1* siRNA, *THBS1* siRNA and/or *CCN1* siRNA for 72 hours, as indicated in the graph legend. Control cells were treated with non-targeting control (NTC) siRNA. Media was collected on the day of transfections and 1, 2 and 3 days following transfection, and cell death was analysed using LDH assay by measuring optical density (OD) at 490 nm and 680 nm. Co-knockdown of PKD1 and THBS1 or PKD1 and CCN1 resulted in rescued levels of cell death compared to PKD1 knockdown on its own. N=5 experiments from 4 HAoEC donors. Differences between groups were analysed using non-parametric Mann-Whitney test (A-B) or two-way ANOVA with Dunnett's post-hoc test vs *PKD1* knockdown embryos (C) and p-values are shown in the graph.

Supp. Figure 16

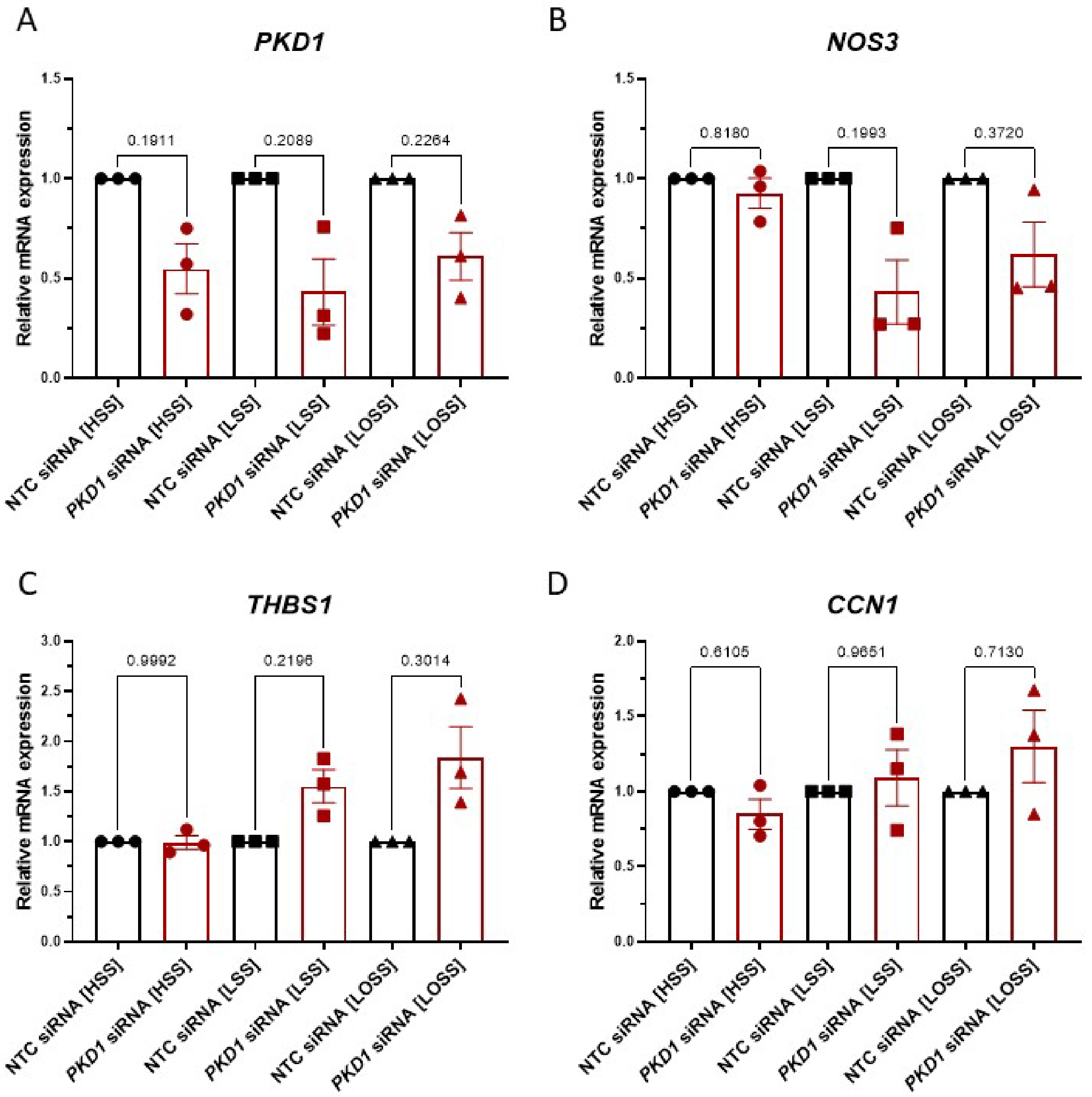

Supp. Fig. 16: Silencing of *PKD1* under flow conditions. Human aortic endothelial cells (HAoECs) were treated with 10 nM *PKD1* siRNA or with non-targeting control (NTC) siRNA prior to exposure to flow using microfluidic pump system (ibidi). The cells were exposed to high laminar shear stress (HSS; 13 dyne/cm^2) – undisturbed flow, and two different types of disturbed flow: low laminar shear stress (LSS; 4 dyne/cm^2) or low oscillatory shear stress (LOSS, ± 4 dyne/cm^2, 0.5 Hz) for 72 hours. Expression of *PKD1*, *NOS3*, *THBS1* and *CCN1* was measured by qPCR (n=4 donors) using *HPRT* as a housekeeping gene. N=3 HAoEC donors.

Supp. Figure 17

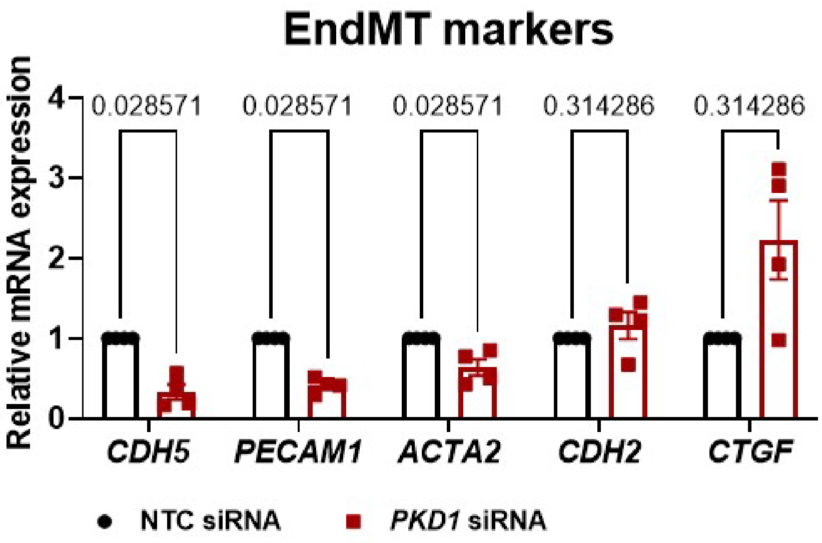

Supp. Fig. 17: Expression of endothelial-to-mesenchymal transition (EndMT) markers in *PKD1*-silenced endothelial cells. HAoECs were transfected with gene-specific *PKD1* siRNA or non-targeting control (NTC) siRNA, and gene expression of selected endothelial (*CDH5, PECAM1*) and mesenchymal markers (*ACTA2, CDH2, CTGF*) was measured by quantitative real-time PCR 72 hours following transfection. Statistical significance was determined using non-parametric Mann-Whitney test. N=4 HAoEC donors.

Supp. Figure 18

A

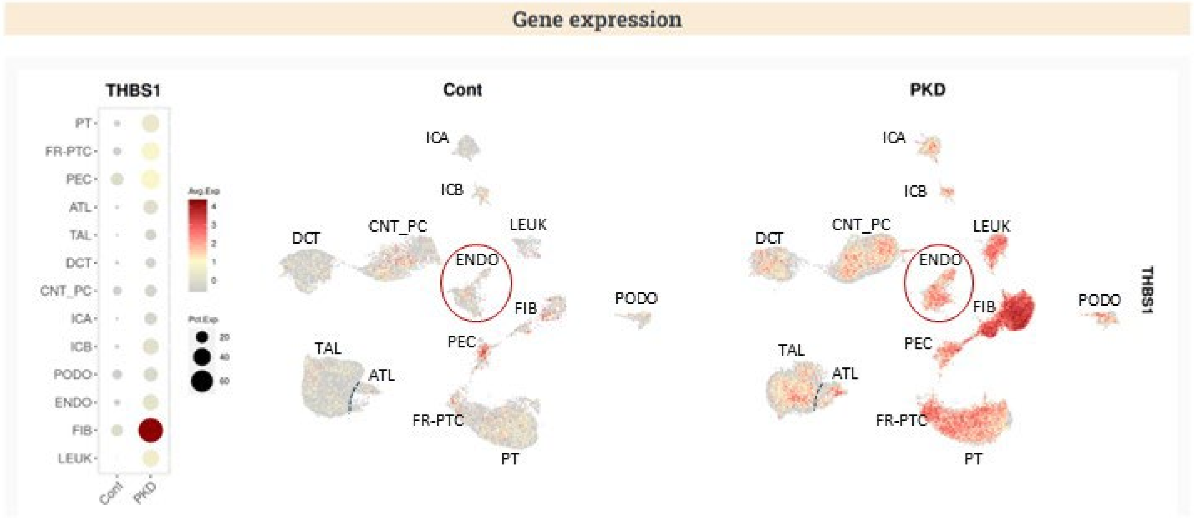

B

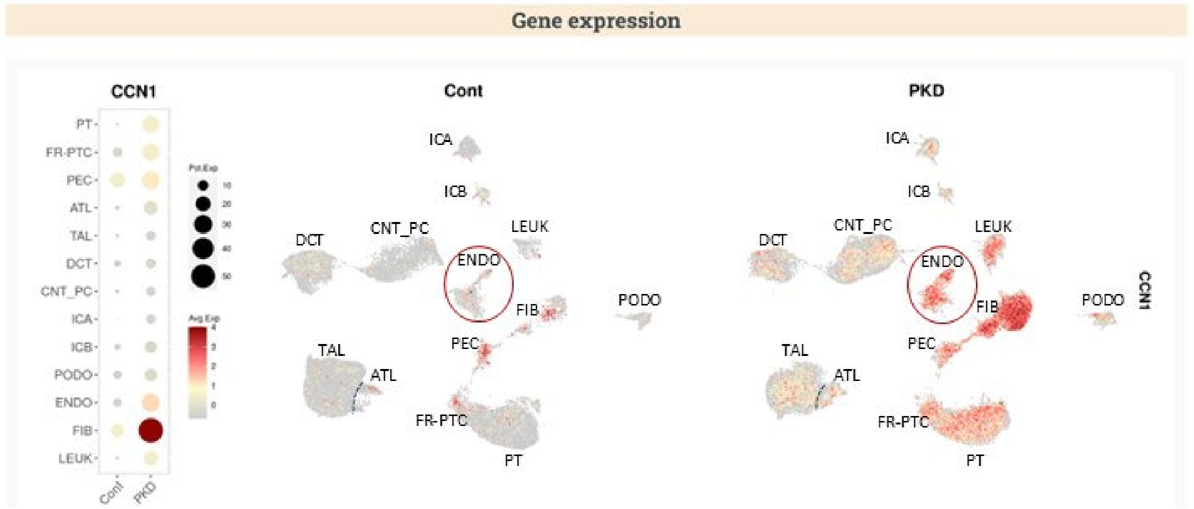

Supp. Fig. 18. Analysis of single nucleus RNA sequencing (snRNAseq) data from the study by Muto et al, 2022 (PMID: 36310237) using Kidney Transcriptomic Interface from the Humphreys lab (https://humphreyslab.com/SingleCell/). A-B THBS1 (A) and CCN1 (B) were both upregulated in the endothelial cell cluster (ENDO) of ADPKD patient compared to controls, as indicated on the diagrams. THBS1 and CCN1 were also upregulated in other cell clusters, such as fibroblasts (FIB), leukocytes (LEUK) and proximal tubule cells (PT).

Supp. Table 1. Zebrafish primers for measuring mechanoreceptor gene expression by qPCR.

Organism	Gene	Forward primer (5'-3')	Reverse primer (5'-3')
Danio rerio	*bact2*	AGCCATCCTTCTTGGGTATG	TGATCTCCTTCTGCATCCTG
Danio rerio	*bdkrb2*	ACGTAATCGTGGGCAAGAAC	TGTCGAATCCGCTTTCTTCT
Danio rerio	*cav1*	CGATGTGGTGAAGGTGGACT	TGGTTACTGTGAAGGTGGTGA
Danio rerio	*cdh5*	TACGCAAGGACTGGAATGAG	GGATTACGATTACCAGGATGG
Danio rerio	*gpc1a*	CACCAAAGCCAGTTACCTCC	AGTGATGTCGATCTCCACCT
Danio rerio	*gpc1b*	GTTCAAGAGCTTCGACGAGTAT	ACACCTTCGACATCTGGGAG
Danio rerio	*itga5*	CCCGTCCTATTGTGAACACC	TATGGCGCAGACTTTCTCCT
Danio rerio	*itgb1a*	AAAACCTGGGGACGAGTCTG	GAGCCAAAACCTATCCTGAAGT
Danio rerio	*itgb1b*	CCAACGACTGCTGGTTCTTC	AGGGACACTCAAGCTCCTC
Danio rerio	*itgb1b.1*	GTGTGGAGTCTGCAGGTGTA	TTTGCATCCAGATCATCCGC
Danio rerio	*itgb1b.2*	GCGAGGAGGTGGAGATCTTT	CGACCTTCATTACACCTGCAG
Danio rerio	*pecam1*	AGCTAAAGTGCCTGTTTCAGTG	GAAGCGTTCCGTTCTTAGCC
Danio rerio	*piezo1*	CAGCTCATGGGAGACGCTAT	ATGATGAAGACGCCCAAATC
Danio rerio	*pkd1a*	CTTCTGGTGGTGGTTTGGG	TCACGAATCCCCACCATGTT
Danio rerio	*pkd1b*	GTCGGCAAGCATCACCTTAG	ATCATCCATCCACCCAGAGC
Danio rerio	*pkd2*	ACGAGTATCCCACAACACTCT	TTCTGCCTCTTCGTGCTGTA
Danio rerio	*sdc4*	GTTTTCGCTGAATCGGTACG	GTCGGTGAACTCAAAGTCTTCA
Danio rerio	*trpv4*	CCATGTTTGACTACGGCACA	CCGATCTCTGCCTTCCCTC

Supp. Table 2. Zebrafish primers for generating whole-mount in situ hybridization probes.

**Organism**	**Gene**	**Forward primer (5’-3’)**	**Reverse primer (5’-3’)**
Danio rerio	*itgb1a*	CGGGTCCAGATATCATCCCC	AACTGCACTGTGGCCTTAAC
Danio rerio	*itgb1b*	CCTCCGAGGTGATTCTGGAA	TCACAAGTCGGACCCTGAAA
Danio rerio	*itgb1b.1*	GAGATGGAAAGCTTGGTGGC	CGCTGTATCTCTCCTCTGGG
Danio rerio	*itgb1b.2*	ACACAACTTCAGGAGGAGCC	CATTCCTCCAGCCAATCACG
Danio rerio	*pkd1a*	ACCTCAAGACCTTCAGCCTC	TGGGGTTTGTGGTAGTGTGT
Danio rerio	*pkd1b*	GCCCAGTGATCAGATGACCT	ATGGGGTCATTACATCGGCT

Supp. Table 3. Morpholino oligo (MO) sequences and concentrations.

MO name	MO sequence (5'-3')	MO position	MO dose	Reference
Control	CCTCTTACCTCAGTTACAATTTATA	N/A	Variable	GeneTools[1]
cav1	AGTCCACCTGGAGGTATAAAAAGAT	i2e3	3 ng	This paper
cdh5	GCACATGCTTGTGTTAGTCTGTTAC	e3i3	3 ng	This paper
itga5	GCATCTGATTAGTGAGACTCACCCA	e2i2	3 ng	This paper
pecam1	ACTGCAAAAGTGTGTATCCTTACAC	e1i1	3 ng	This paper
piezo1	ATCCGCATACCACCTGAAGCTCCAT	ATG	3 ng	Faucherre et al[2]
pkd1a	ACCATCACAGAACGTACCAGAAGGT	e2i2	3 ng	This paper
pkd2	AGGACGAACGCGACTGGAGCTCATC	ATG	3 ng	Sun et al[3]
sdc4	TAACAGTGTGTCCTCACCTGCAAGC	e3i3	3 ng	This paper
sih	CATGTTTGCTCTGATCTGACACGCA	ATG	1 ng	Sehnert et al[4]

Supp. Table 4. Zebrafish primers for validating morpholino oligo efficiency using RT-PCR and qPCR.

Organism	Gene	Forward primer (5'-3')	Reverse primer (5'-3')
Danio rerio	*bact2*	TGAAATTGCCGCACTGGTTGTTG	ATGGCGGGGGTGTTGAAGGTC
Danio rerio	*cav1*	GCTCACTCGCCGTTTATCAG	ACCCTTCACTTCTGCTCACA
Danio rerio	*cav1*	TGGATAACGACAGCATCAACG	AAGTCCACCTTCACCACATC
Danio rerio	*cdh5*	GGTTCACATGCAGCTGGAAA	AGTGGAGGAATCATCAGCGT
Danio rerio	*cdh5*	TCCTCTAGAGCAGGAACAACT	TTTTGAAGAGGTCTGTGCCA
Danio rerio	*itga5*	TTTGGGAGCTCCATTACACC	TGTCTTCACCACCAAATGGA
Danio rerio	*pecam1*	AATCTGCGTCACGTTCACCT	TGACCTCCTGATTGTCCTCG
Danio rerio	*pkd1a*	CTTCGTTCAGGTGGACAGTG	TGTGAGTGTATGCGTGAGGA
Danio rerio	*sdc4*	CTGACGATGAGGATGGCTTT	TCACATCTGCAAACCCAGAG

Supp. Table 5. Mouse genotyping primers.

Organism	Gene	Forward primer (5'-3')	Reverse primer (5'-3')
Mus musculus	*Pkd1*	CCTGCCTTGCTCTACTTTCC	AGGGCTTTTCTTGCTGGTCT
Mus musculus	*Pkd2*	GGGGTTTCCTATGAAGAGTTCCAAG	CTGACAGGCACCTACAGAACAGTG
Mus musculus	*Cre*	TCGATGCAACGAGTGATGAG	AGTGCGTTCGAACGCTAGAG

Supp. Table 6. Mouse and human qPCR primers.

Organism	Gene	Forward primer (5'-3')	Reverse primer (5'-3')
Mus musculus	*Acta2*	CATCATGCGTCTGGACTTGG	AATCTCACGCTCGGCAGTAG
Mus musculus	*Cdh5*	TCTTGCCAGCAAACTCTCCT	TTGGAATCAAATGCACATCG
Mus musculus	*Hprt*	AGTCCCAGCGTCGTGATTAG	TCTCGAGCAAGTCTTTCAGTCC
Mus musculus	*Pecam1*	CGGTGTTCAGCGAGATCC	ACTCGACAGGATGGAAATCAC
Mus musculus	*Pkd1*	CACTGGTGGAGCTGGACTTA	TTGCACTCAAATGGGTTCCC
Mus musculus	*Pkd2*	TACGGCATGATGAGCTCCAA	AAGGAGCCTTCGGTGAACTT
Homo sapiens	*ACTA2*	CAAGTGATCACCATCGGAAAT	GACTCCATCCCGATGAAGGA
Homo sapiens	*CCN1*	CAGTTGGAAAAGGCAGCTCA	TCTTTGAGCACTGGGACCAT
Homo sapiens	*CDH2*	GCACAGATGTGGACAGGATT	CAGCACAAGGATAAGCAGGA
Homo sapiens	*CDH5*	CACCTTCTGCGAGGATATGG	AGGAAGATGAGCAGGGTGAT
Homo sapiens	*CTGF*	GGAAGAGAACATTAAGAAGGGCA	CACAAGGGCCTATTCTGTCA
Homo sapiens	*HPRT*	TTGGTCAGGCAGTATAATCC	GGGCATATCCTACAACAAAC
Homo sapiens	*NOS3*	TGAAGCACCTGGAGAATGAG	TTGACCATCTCCTGATGGAA
Homo sapiens	*PECAM1*	GAGTCCAGCCGCATATCC	TGACACAATCGTATCTTCCTTC
Homo sapiens	*PKD1*	CCTCCTGGTGTACATGCTTT	TCCTCAGACCGCGTGATG
Homo sapiens	*PKD2*	CCAAGATTGACGCCGTGATC	CTTTCATCCTCGGCCACCC
Homo sapiens	*THBS1*	CTGTCAGAACTCAGTTACCATCT	CGAGGACAGCATTCTCCATC

Supp. Table 7. Antibodies

Antibody	Source	Catalogue number	Use	Final concentration
ACTA2	DAKO	M0851	Immunohistochemistry	5 μg/ml
Active caspase 3	R&D Systems	AF835	Zebrafish immunofluorescence staining	5 μg/ml
Active caspase 3	Cell Signaling	9661	Mouse en face and HAoEC immunofluorescence staining	2 μg/ml
BMP4	Santa Cruz	sc-6896	Mouse en face immunofluorescence staining	5 μg/ml
CCN1	Thermo Fisher	PA5-78022	Mouse en face immunofluorescence staining	2.6 μg/ml
CDH5	BD Pharmingen	555289	Mouse en face immunofluorescence staining	5 μg/ml
CDH5	BD Pharmingen	555661	HAoEC immunofluorescence staining	5 μg/ml
eNOS	Cell Signaling	32027	Mouse en face immunofluorescence staining	5 μg/ml
			Western Blotting	0.5 μg/ml
PDHX	Santa Cruz	sc-393644	Western blotting	0.2 μg/ml
THBS1	abcam	ab267388	Mouse en face immunofluorescence staining	5 μg/ml
TXNDC5	Proteintech	19834-1-AP	Mouse en face immunofluorescence staining	2.5 μg/ml

Supp. Table 8. Reagent and chemical catalogue number

Reagent/chemical	Source	Catalogue number
2-Mercaptoethanol	Sigma-Aldrich	BCCD3726
2-Propanol	Sigma-Aldrich	I9030
4x Laemmli Sample Buffer	Bio-Rad	1610747
Agarose	Thermo Fisher	16500500
BioMix Red	Bioline	BIO-25006
Bovine Serum Albumin	Sigma-Aldrich	A9647 - 50G
Click-iT™ TUNEL Colorimetric IHC Detection Kit	Invitrogen	C10625
Collagenase type I	SLS	C1-28-100MG
Collagenase type XI	VWR International	C7657-25MG
CyQUANT LDH Cytotoxicity Assay	Thermo Fisher Scientific	C20300
DAB Substrate Kit, Peroxidase (HRP)	Vector Labs	SK-4100
DAPI	Sigma-Aldrich	D9542
Deoxyribonuclease	Cambridge Bioscience	T64750
dNTP Mix (25 mM each), Quantity: 1mL	Thermo Fisher	R1121
DPX Mountant for histology	Sigma-Aldrich	6522
ECL Select™ Western Blotting Detection Reagent	Cytiva	RPN2235
Elastase	Promega UK Ltd	V1891
Endothelial Cell Growth MV2	PromoCell	c-22022
Ethidium bromide	Merck	E1510-10ML
Formaldehyde (4%)	VWR International	J60401.AP
Gelatin from bovine skin	Sigma-Aldrich	9000-70-8
Glycine	Fisher Scientific	G/0800/60
H&E Staining Kit	abcam	ab245880
Heparin sodium salt	Sigma-Aldrich	H3393

Supp. Table 8. Reagent and chemical catalogue number (continued)

Reagent/chemical	Source	Catalogue number
Hyaluronidase	VWR International	P5233
iScript™ cDNA Synthesis Kit	Bio-Rad	1708891
Isopropanol, Extra Pure	Fisher Scientific	11428782
Lipofectamine RNAiMAX Transfection Reagent	Thermo Fisher Scientific	13778150
Mini-PROTEAN TAX Gels	Bio-Rad	4561093
miRNeasy Mini Kit	QIAGEN	217084
Nitric Oxide Assay	Sigma-Aldrich	MAK454
Normal Goat Serum	abcam	ab7481
Oil Red O	Thermo Fisher	A12989.14
Phosphate Buffered Saline (Dulbecco A)	Oxoid	BR0014G
Picrosirius Red	abcam	ab150681
Precision Plus Protein Dual Color Standards	Bio-Rad	1610374
ProLong Gold	Thermo FIsher	P36930
QIAzol Lysis Reagent	QIAGEN	79306
Quick-Load® Purple Low Molecular Weight DNA Ladder	NEB	N0557S
RNase-Free DNase Set	QIAGEN	79254
RNeasy MINI Kit	QIAGEN	74104
Sodium dodecyl sulphate (SDS)	GE Healthcare Life Sciences	17 - 1313 - 01
SsoAdvanced Universal SYBR Green Supermix	Bio-Rad	1725271
Supplement Mix Endothelial Cell Growth Medium MV2	PromoCell	C - 39226
Taq DNA polymerase	QIAGEN	201203
TO-PRO-3	Fisher Scientific	10710194
TRIS - buffered saline (TBS, 10X, pH 7.6)	Thermo Scientific	JB2662.K7
Triton X-100	Thermo Fisher Scientific	A16046-AE

Supp. Table 8. Reagent and chemical catalogue number (continued)

Reagent/chemical	Source	Catalogue number
Triton X-100	Thermo Fisher Scientific	A16046-AE
Trizma® base	Sigma-Aldrich	1003337800
TruStain FcX PLUS	BioLegend UK Ltd	156604
Trypsin	Fisher Scientific	25200072
Tween® 20	Fisher Scientific	BP337 - 500

Supp. Table 9. HAoEC PromoCell Lot numbers

HAoEC Donor	Lot number
1	434Z005.1
2	472Z002.1
3	445Z040
4	483Z027.6

Supplementary references:

1 *Gene Tools Negative control morpholino oligo*, <https://www.gene-tools.com/content/negative-control-morpholino-oligos>

2 Faucherre, A., Kissa, K., Nargeot, J., Mangoni, M. E. & Jopling, C. Piezo1 plays a role in erythrocyte volume homeostasis. *Haematologica* **99**, 70-75, doi:10.3324/haematol.2013.086090 (2014).

3 Sun, Z. *et al.* A genetic screen in zebrafish identifies cilia genes as a principal cause of cystic kidney. *Development* **131**, 4085-4093, doi:10.1242/dev.01240 (2004).

4 Sehnert, A. J. *et al.* Cardiac troponin T is essential in sarcomere assembly and cardiac contractility. *Nat Genet* **31**, 106-110, doi:10.1038/ng875 (2002).
